# Next-generation immunotherapy biologics for glioblastoma

**DOI:** 10.3389/fimmu.2026.1775093

**Published:** 2026-03-18

**Authors:** Ethan Schonfeld, Adam Sjoholm, Joe Ha, Justin Liu, George Nageeb, Shreyas Annagiri, Lily Kim, John Choi, Michael Lim

**Affiliations:** 1 Stanford University School of Medicine, Stanford University, Stanford, CA, United States; 2 Stanford School of Humanities and Sciences, Stanford University, Stanford, CA, United States; 3 Department of Neurosurgery, Stanford University School of Medicine, Stanford University, Stanford, CA, United States

**Keywords:** biologics, cancer vaccination, CAR (chimeric antigen receptor) T cells, checkpoint inhibition, glioblastoma, immunotherapy, myeloid derived suppressor cells (MDSCs), oncolytic virus

## Abstract

Glioblastoma (GBM) remains largely resistant to immunotherapy despite the success of immune checkpoint inhibitors in other solid tumors. Phase III trials have not demonstrated survival benefit for anti-PD-1 monotherapy, likely reflecting the GBM tumor microenvironment’s profound myeloid-driven immunosuppression, low neoantigen burden, intratumoral heterogeneity, and adaptive resistance. These challenges have driven the development of next-generation immunotherapy biologics designed to reprogram the tumor microenvironment and overcome the innate and adaptive resistance of GBM. This review synthesizes advances in immunotherapy biologics including immune checkpoint combinations, cytokine and immunomodulatory proteins, adoptive cell therapies, vaccines, and oncolytic viruses, highlighting key preclinical insights and emerging clinical trial results. We conclude that improved tumor subtyping and immune profiling will be crucial to guide combination strategies that may achieve durable clinical benefit in GBM.

## Introduction

Immunotherapy biologics are redefining cancer therapy and include immune checkpoint inhibitors (ICIs), adoptive cell therapies, oncolytic viruses, vaccines, cytokine and chemokines, and myeloid modulating biologics. However, while anti-PD-1 has become first-line treatment for many cancers (1, 2), anti-PD-1 did not have an overall survival (OS) benefit in newly diagnosed or recurrent glioblastoma (GBM) (3–5). GBM, the most common primary malignant brain tumor, has a median survival of 15–18 months (6). The standard of care for primary GBM has not changed since 2005 and is comprised of maximal safe resection plus adjuvant temozolomide (TMZ) and external beam radiation (7).

GBM has multiple unique features from other solid tumors that explain its poor clinical outcomes and resistant to therapies. Unlike most other solid tumors, the blood brain barrier (BBB) selectively prohibits most small molecule drugs and effectively all biological agents from reaching the tumor. The immunogenicity of GBM does increase BBB permeability; however, this effect is unequally spatially distributed across the tumor and therefore restricts drug delivery to invasive margins on the periphery (8). Like other solid tumors, angiogenesis is a dominant program; however, unlike in other tumors, GBM angiogenesis utilizes multiple mechanisms other than VEGF, resulting in extreme heterogeneity of the vascular compartment (9). Another unique feature to GBM is its invasive growth via glial interactions. Oligodendrocytes secrete CCL5, the chemokine to the CCR5 receptor that is enriched on GBM tumor cells, promoting tumor growth, stemness, and invasiveness (10). In addition to oligodendrocytes, microglia in GBM mobilize early, forming nets around invasive tumor tissue and function to guide migration. Myeloid cells have further pro-tumoral effects such as restructuring the extracellular matrix and driving immunosuppression (11, 12). This immunosuppression results in a GBM tumor microenvironment (TME) where infiltrating T cells don’t exhibit transcriptional exhaustion states as found in other solid tumors but rather uniquely suppressed transcriptional programs (13). Collectively, these structural and microenvironmental features create a permissive niche for both tumor-intrinsic immune evasion and extrinsic immune suppression.

GBM resistance can be categorized into tumor cell–intrinsic and tumor-extrinsic microenvironmental mechanisms. Intrinsically, GBM tumor cells have high intratumoral heterogeneity and a low neoantigen load, making the tumor of low immunogenic quality (14). Immunosuppression is further intrinsically accomplished via the expression of immunosuppressive molecules including PD-L1 a checkpoint ligand, IDO1 which depletes tryptophan to promote myeloid derived suppressor cells (MDSCs) and inhibit effector T and NK cells, IL-10 to increase Tregs and promote T cell exhaustion, and TGF-β whose secretion reduced CD8+ T cell and NK cell cytotoxicity (15). The production of IL-10, TGF-β, as well as lactate and hypoxic conditions by tumor cells polarize myeloid cells to immunosuppressive states (16, 17). Glioma stem cells (GSCs) have complex interactions with surrounding immune cells, producing chemokines that attract pro-tumoral myeloid cells (e.g.: E-MDSCs) to the TME (18). However, a significant resistance of GBM comes from extrinsic mechanisms, which are largely myeloid driven.

The dominant extrinsic mechanism of resistance in GBM is innate myeloid–driven immunosuppression. Myeloid cells in the GBM TME include myeloid-derived suppressor cells (MDSCs), M2 polarized tumor associated macrophages (TAMs), and microglia. These myeloid compartments are further split into specific pro-tumor roles. MDSCs include E-MDSCs and M-MDSCs, where M-MDSCs are derived from E-MDSCs and appear to function in states of hypoxia and cell stress (18). E-MDSCs function to potentiate tumor growth and immunosuppression but can transition to other MDSC states that alternatively promote tumor growth and immunosuppression when targeted. Therefore, targeting of only one MDSC subpopulation may not inhibit their pro-tumor effects, instead likely requiring whole MDSC compartment targeting. Single-cell and spatial transcriptomic profiling of adult IDH-wild-type GBM shows that GSC driven infiltration (19) of heterogeneously polarized, immunosuppressive myeloid cells can make up roughly half of all tumor‐resident cells while tumor-infiltrating lymphocytes are typically <5% of the immune infiltrate (18, 20–22). Myeloid cell immunosuppression is multifaceted, comprising metabolic, checkpoint, cytokine, among other mechanisms. Myeloid derived metabolic immunosuppression includes arginine depletion which results in T cell anergy, as well as myeloid tryptophan catabolism and adenosine production resulting in T cell dysfunction and increased Tregs (23). Immune checkpoints, expressed on myeloid cells (PD-L1/PD-1, Galectin-9/TIM-3, VISTA/CD28) contribute to GBM immunosuppression via myeloid polarization and decreased effector T cell function (24–27). Anti-tumor immune function is further reduced in the GBM TME by the downregulation of MHC-II on myeloid cells [e.g.: TLR2 activation (28)], phagocytosis suppression [e.g.: CD47 overexpression (29)], and extracellular matrix (ECM) remodeling [e.g.: TAMs enhance MMP signaling (30)] with perivascular myeloid cuffs and hypoxic regions. In addition to myeloid-mediated immunosuppression, emerging evidence suggests that mesenchymal stromal populations and fibrosis-like ECM remodeling contribute to GBM resistance and immunosuppression. Perivascular fibroblasts have been correlated with both poor survival and poor ICI response in GBM (31). Following myeloid targeting therapy, myeloid and TGF-β signaling promoted a fibrotic treatment response by perivascular fibroblasts that reduced immune surveillance and enhanced tumor cell survival (32). Inhibition of the fibrotic adaptive response improved response to myeloid targeting therapy in GBM, demonstrating the importance of fibrosis adaptive resistance mechanisms in GBM.

**Figure 1:**
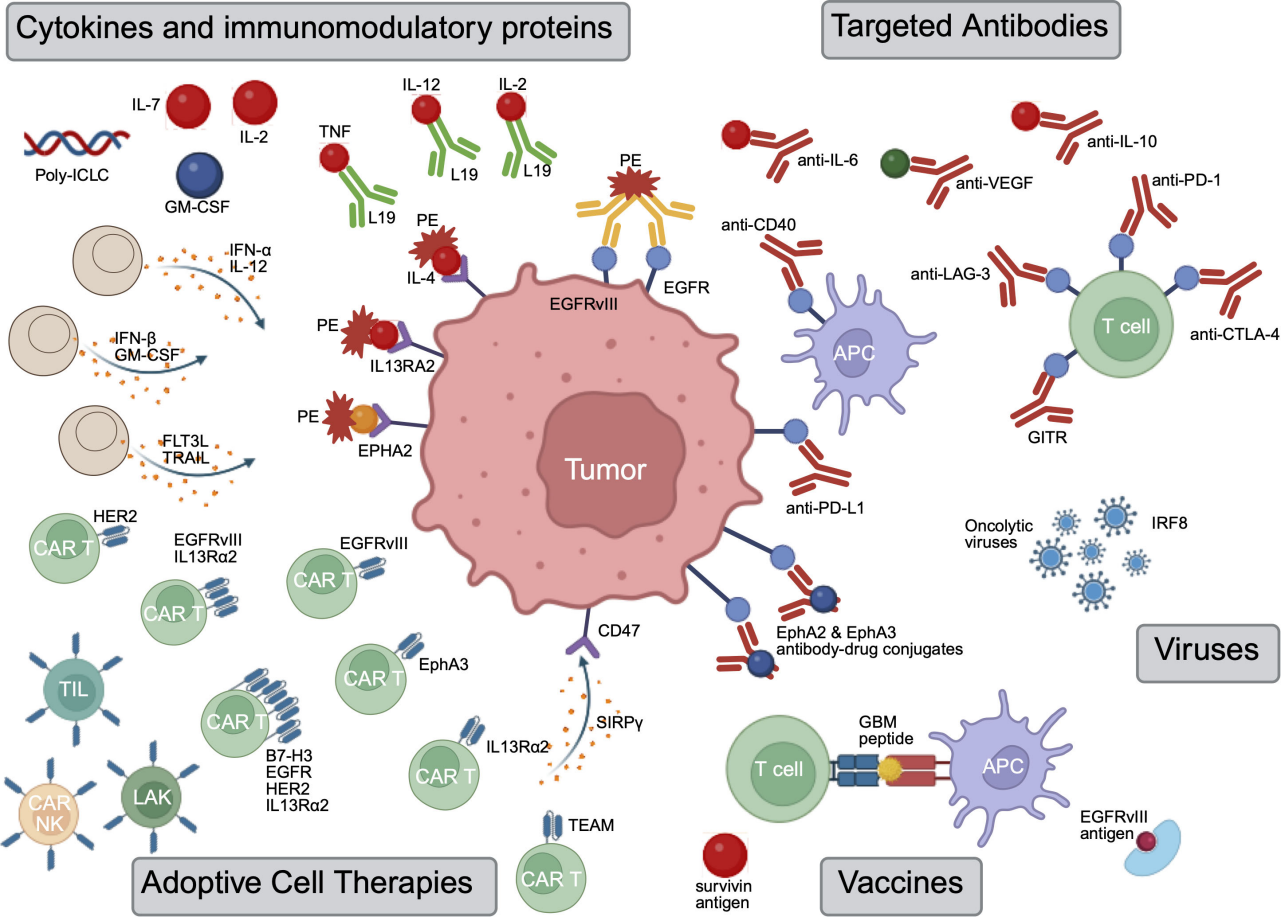
An overview of the principal biologic strategies discussed in this review and their interaction with tumor cells and immune elements within the glioblastoma microenvironment. The schematic highlights three major domains: (1) immune checkpoint and antibody-based strategies targeting T cells and myeloid cells, (2) cytokine and immunomodulatory protein delivery platforms, and (3) adoptive cell and vaccine-based approaches directed against tumor-associated antigens. The figure emphasizes the multidirectional crosstalk between tumor cells, myeloid populations, lymphocytes, and engineered biologics within the GBM tumor microenvironment. The diagram is conceptual and does not represent relative dominance or sequence of signaling events.

Upon therapy, GBM exhibits adaptive immunosuppression including a cytokine driven increase across multiple innate immunosuppression mechanisms, the up-regulation of alternate checkpoints such as TIM-3 and TIGIT in response to ICI (33), secretion of tumor derived extracellular vesicles that inhibit T cell clonal expansion (34), and upregulation of neutrophil influx and mesenchymal state following myeloid depletion (35). With the presence of multiple such immunosuppressive mechanisms, no trial of any ICI for GBM has demonstrated improved survival. Thus, ICI therapies are increasingly being combined with recently developed immunotherapy biologics (36). To orient the reader to the breadth of biologic strategies under investigation, **Figure 1** provides a high-level schematic of therapeutic entry points into the GBM microenvironment, which are subsequently discussed in focused sections throughout the manuscript. As preclinical research continues to discover and characterize the major axes that govern GBM immunosuppression, immunotherapy biologics are being developed and translated, often in combined immunotherapies, for GBM (**Table 1**).

**Table 1:** Results from phase II and III clinical trials of immunotherapy in patients with glioblastoma. For inclusion studies were identified from clinicaltrials.gov using the search: glioblastomaimmunotherapy Phase: 2, 3 Interventional studies Studies with results Study start from 01/01/2012 to 02/03/2026.

NCT	Enrollment	Immunotherapeutic agent(s)	Patient population	Overall survival (OS)	Progression free survival (PFS)	Objective response rate (ORR)
NCT02078648	74	Arm 1: SL-701 (glioma antigen vaccine), vaccine adjuvants (imiquimod, GM-CSF)	Recurrent GBM	OS at 12 Months: 20/46 (43.5%)	1.9 months, 95% CI: 1.8-2.7)	2.2%, 95% CI: (0.1 to 9.9)
		Arm 2: SL-701 (glioma antigen vaccine), poly-ICLC, Bevacizumab (anti-VEGF)		OS at 12 Months: 14/28 (50%)	5.6 months, 95% CI: (3.6-10.0)	14.3%, 95% CI: (5.0 to 29.8)
NCT02336165	159	Radiation therapy followed by Durvalumab (anti-PD-L1)	Newly Diagnosed Un-methylated GBM	Up to 36 months: 64.8 weeks (n=40)	19.9 weeks, 95% CI: (16.0-32.3)	NR
		Durvalumab (anti-PD-L1) and Bevacizumab (anti-VEGF)	Recurrent GBM – bevacizumab naïve	37.3 weeks (n=33)	16.0 weeks, 95% CI: (11.9-16.1)	
NCT04013672	41	Pembrolizumab (anti-PD-1), SurVaxM (survivin peptide vaccine), GM-CSF, montanide	Recurrent GBM – immunotherapy naïve	NR	PFS-6: 34.5%, 95% CI: (19.8-49.6)	NR
			Recurrent GBM – failed prior immunotherapy		NR	
NCT02858895	47	MDNA55 (IL-4 fused to Pseudomonas aeruginosa exotoxin A)	Recurrent or Progressive GBM	10.2 months (n=47, ITT analysis) 11.64 months (n=44, PP analysis)	3.61 months (95% CI: 2.79-5.08)	2.4%
NCT01454596	18	EGFRvIII CAR-T, IL-2	Recurrent Glioblastoma	NR	2.7 months (95% CI: 0.9-12.5) (n=3) (note: Cohort 6 results shown, using 3x10(9) cells)	0%
NCT03149003	221	Arm 1: DSP-788 (WT1 peptide vaccine) Dosing Emulsion + Bevacizumab (anti-VEGF)	Recurrent or Progressive GBM	OS at 12 months; 37.9% (95% CI: 28.7-47.0)	5.3 months (95% CI: 3.9-5.6)	21.1% (95% CI: 13.9-30.0)
		Arm 2: Bevacizumab (anti-VEGF)		OS at 12 months; 31.6% (95% CI: 22.9-40.7)	3.8 months (95% CI: 3.7–5.6)	13.0% (95% CI: 7.3-20.8)
NCT03367715	10	Nivolumab (anti-PD1) and Ipilimumab (anti-CTLA-4) followed by radiation therapy	MGMT Un-methylated Glioblastoma	OS at 3 years; 16.85% (95% CI: 4.49-32.89)	5.92 months (95% CI: 1.48-13.93)	NR
NCT04406272	15	Arm 1: VB-111 (adenoviral vector) before and after surgery	Recurrent or Primary Glioblastoma	OS at 11–15 months; 0 participants	PFS at 6 months; 1 participant	NR
		Arm 2: VB-111 (adenoviral vector) after surgery		OS at 11–15 months; 1 participant	PFS at 6 months; 1 participant	
		Arm 3: Placebo		OS at 11–15 months; 1 participant	PFS at 6 months; 0 participants	
NCT01811498	31	Superselective Intra-arterial Cerebral Infusion of Bevacizumab (anti-VEGF)	Newly Diagnosed GBM	Median OS; 23.1 months (95% CI: 12.2-36.9)	PFS at 6 months; 91.3 participants (95% CI: 69.5-97.9)	NR
NCT02663271	10	Optune Tumor Treating Fields + Bevacizumab (anti-VEGF)	Bevacizumab – Refractory GBM	Insufficient number of participants with events	NR	NR
NCT03018288	90	Arm 1: Heat Shock Protein Peptide Complex-96 vaccine + Pembrolizumab (anti-PD-1) + RT/TMZ	Newly Diagnosed GBM	OS at 12 months; 52.5% (95% CI: 24.5-100)	NR	Complete Response: 0% (95% CI: 0-60.2)
		Arm 2: Pembrolizumab (anti-PD-1) + RT/TMZ		OS at 12 months; 71.4% (95% CI: 44.7-100)		Complete Response Rate: 0% (95% CI: 0-45.9)
NCT03452579	90	Arm 1: Nivolumab (anti-PD1) + Standard Dose Bevacizumab (anti-VEGF)	Recurrent Glioblastoma	OS at 12 months; 41.1% (95% CI: 26.1-56.1)	PFS at 6 months; 46.7% (95% CI: 32.1-61.2)	Overall Response Rate; 18%
		Arm 2: Nivolumab (anti-PD1) + Low Dose Bevacizumab (anti-VEGF)		OS at 12 months; 37.7% (23.1-52.4)	PFS at 6 months: 44.6% (95% CI: 29.4-59.8)	Overall Response Rate; 16%
NCT02337491	80	Arm 1: Pembrolizumab (anti-PD-1) + Bevacizumab (anti-VEGF)	Recurrent Glioblastoma	8.8 months (95% CI: 7.7-14.2)	4.1 months (95% CI: 2.8-5.5)	Overall Radiographic Response; 0.20% (95% CI: 0.11-0.32)
		Arm 2: Pembrolizumab (anti-PD-1)		10.3 months (95% CI: 8.5-12.5)	1.4 months (95% CI: 1.4-2.7)	Overall Radiographic Response; 0% (95% CI: 0.0-0.095)
NCT02366728	64	Human CMV pp65-LAMP mRNA pulsed autologous DC vaccine	Newly Diagnosed Glioblastoma	16 months (95% CI: 12.8-25.5)	6.5 months (95% CI: 4.4-12.1)	NR
NCT03047473	30	Avelumab (anti-PD-L1)	Newly Diagnosed Glioblastoma	NR	NR	NR
NCT03797326	611 (n=102 GBM)	Lenvatinib + Pembrolizumab (anti-PD-1)	Patients with previously treated solid tumors	8.6 months (95% CI: 7.4-10.8)	3.0 months (95% CI: 2.9-4.1)	21.8% (95% CI: 14.2-31.1)
NCT02465268	175	Pp65-shLAMP dendritic cells with GM-CSF	Newly Diagnosed Glioblastoma	16.9 months (95% CI: 12.7-22.8)	6.1 months (95% CI: 3.9-11.0)	NR
		Pp65-flLAMP dendritic cells with GM CSF		16.2 months (95% CI: 13.8-19.2)	6.4 months (95% CI: 4.2-10.8)	
NCT03405792	40	Optune Tumor Treating Fields + Pembrolizumab (anti-PD-1) + TMZ	Newly Diagnosed Glioblastoma	NR	11.79 months (95% CI: 8.71-22.72)	NR
NCT04225039	39	INCMGA00012 (anti-PD1) + INCAGN01876(anti-GITR) + SRS	Recurrent Glioblastoma	NR	NR	Objective Radiographic Response; 0% (95% CI: 0-19)
NCT02017717	369	Arm 1: Nivolumab (anti-PD1)	Recurrent Glioblastoma	OS at 12 months; 41.8% (95% CI: 34.7-48.8)	1.51 months (95% CI: 1.48-1.61)	7.8% (95% CI: 4.1-13.3)
		Arm 2: Bevacizumab (anti-VEGF)		OS at 12 months; 42.4% (95% CI: 34.9-49.6)	3.61 months (95% CI: 2.99-4.60)	23.1% (95% CI: 16.7-30.5)
NCT03661723	60	Arm 1: Pembrolizumab (anti-PD-1) + RT	Bevacizumab Naïve and Bevacizumab Resistant Recurrent Glioblastoma	OS at 12 months; 40% (95% CI: 22.8-56.6)	4.2 months (SE: 0.4551)	1 participant
		Arm 2: Pembrolizumab (anti-PD-1) + Bevacizumab (anti-VEGF) + RT		OS at 12 months; 16.6% (95% CI: 6.0-31.7)	4.0 months (SE: 0.5467)	1 participant
NCT02661282	65 (n=3 for Newly Diagnosed GBM patients in dose level 1 x 10^8)	Dose escalation of CMV-specific T cells + TMZ	Recurrent or Newly Diagnosed Glioblastoma	Median OS for newly diagnosed GBM patients, Dose Level 1 x 10^8; 24 weeks (95% CI: 13-24)	Median PFS for newly diagnosed GBM patients, Dose Level 1 x 10^8; 19 weeks (95% CI: 9-20)	Objective Response Rate for newly diagnosed GBM patients, Dose Level 1 x 10^8: 3 participants
NCT02343549	13	Bevacizumab (anti-VEGF) + Optune Tumor Treating Fields, + TMZ + RT	Newly Diagnosed Unresectable GBM	9.9 months (95% CI: 4.8-12.8)	7.9 months (95% CI: 4.8-10.4)	33.3% (3/9)
NCT03927222	6	Human CMV pp65-LAMP mRNA autologous dendritic cells + GM CSF	Newly Diagnosed WHO Grade IV glioma, CMV positive, MGMT unmethylated	17.4 months (95% CI: 13.3-NA)	NR	NR
NCT04704154	175 (n=30 GBM/Anaplastic Astrocytoma)	Regorafenib (multi-kinase inhibitor) + Nivolumab (anti-PD1)	Solid Tumors	GBM/AA Cohort OS; 245 days (95% CI: 127-377)	GBM/AA Cohort PFS; 55 days (95% CI: 52-84)	GBM/AA Cohort ORR; 1 participant
NCT01738646	48	Vorinostat (histone deacetylase inhibitor) + Bevacizumab (anti-VEGF)	Recurrent Glioblastoma	10.4 months (95% CI: 7.6-12.8)	3.7 months 2.9-4.8	Radiographic Response; 22.5% (95% CI: 12.1-37.7)
NCT02330562	121(n=30 for Marizomib 0.8 mg/m2 + Bevacizumab 10mg/kg cohort)	Dose Escalation of Marizomib (proteasome inhibitor) + Bevacizumab (anti-VEGF)	Recurrent Glioblastoma	OS of Marizomib 0.8 mg/m2 + Bevacizumab 10mg/kg cohort; 8.3 months (95% CI: 4.8-11.3)	PFS of Marizomib 0.8 mg/m2 Bevacizumab 10mg/kg cohort; 1.8 months (95% CI: 1.5-1.9)	Radiographic Objective Response Rate of 0.8 mg/m2 Bevacizumab 10mg/kg cohort; 3.3% (95% CI: 0.1-17.2)
NCT02794883	36	Arm 1: Tremelimumab (anti-CTLA-4)	Recurrent Glioblastoma	7.246 months (95% CI: 2.746-16.32)	NR	NR
		Arm 2: MEDI4736 (anti-PD-L1)		11.71 months (95% CI: 8.332-32.71)		
		Arm 3: Tremelimumab (anti-CTLA-4) + MEDI4736 (anti-PD-L1)		7.703 months (95% CI: 7.411-40.14)		
NCT01582152	12	TPI 287 + Bevacizumab (anti-VEGF)	Recurrent Glioblastoma	NR	12.5 weeks (95% CI: 6-37)	40%
NCT02120287	16	Border Zone SRS + Bevacizumab (anti-VEGF)	Recurrent or Progressive Glioblastoma	11.73 months (95% CI: 8.64-26.05)	PFS at 6 months: 56.2% (95% CI: 29.9-80.2)	NR
NCT01564914	22	Carotuximab (anti-endoglin) + Bevacizumab (anti-VEGF)	Recurrent or Progressive Glioblastoma	5.75 months (95% CI: 4.21–9.86)	1.81 months (1.25-2.07)	NR
NCT01894061	25	Bevacizumab (anti-VEGF) + Nova Tumor Treating Fields	Recurrent Glioblastoma	NR	PFS at 6 months; 33% (95% CI: 9-60)	Complete Response: 1 participant Partial Response: 3 participants
NCT03532295	51	Arm 1: Retifanlimab (anti-PD1) + RT + Bevacizumab (anti-VEGF)	Recurrent Glioblastoma	OS at 12 months; 50% (95% CI: 34-75)	6.98 months (95% CI: 5.55–11.04)	NR
		Arm 2: Retifanlimab (anti-PD1) + RT + Bevacizumab (anti-VEGF) + Epacadostat (ID01 inhibitor)		OS at 12 months; 38% (95% CI: 23-62)	7.20 months (95% CI: 5.78–8.61)	
NCT03139916	36	Bavituximab (anti-phosphatidylserine) + RT + TMZ	Newly Diagnosed Glioblastoma	OS at 12 months; 72.7% (95% CI: 59-90)	6.9 months (95% CI: 6.2-9.7)	Radiographic Response Rate: 4 participants
NCT04006119	40	Ad-RTS-hIL-12 + Veledimex + Cemiplimab (anti-PD-1)	Recurrent or Progressive Glioblastoma	NR	89.0 days (SD: 95.6)	3 participants
NCT03347617	52	Ferumoxytol MRI + Pembrolizumab (anti-PD-1)	Newly Diagnosed Glioblastoma	15.2 months (95% CI: 12.5-17.1)	10.4 months (95% CI: 7.1-11.7)	Complete Response: 8 participants
NCT02968940	6	Avelumab (anti-PD-L1) + Hypofractionated Radiation Therapy	IDH mutant Glioblastoma	10.1 months (95% CI: 6.8–12)	4.2 months (95% CI: 1.4–5.7)	NR
NCT02342379	35	Bevacizumab (anti-VEGF) + TH-302	Glioblastoma with Bevacizumab treatment failure	NR	PFS at 4 months; 32 participants	NR
NCT04479241	25	Lerapolturev (oncolytic viral therapy) + Pembrolizumab (anti-PD-1)	Recurrent Glioblastoma	OS at 24 months; 10.2 months (95% CI: 8.9–15.9)	NR	0 participants
NCT03430791	5	Nivolumab (anti-PD1) + Ipilimumab (anti-CTLA-4) + Optune Tumor Treating Fields	Recurrent Glioblastoma	NR	NR	NR
NCT02986178	121	Lerapolturev (oncolytic viral therapy) + Lomustine	Grade IV malignant glioma	7.1 months (95% CI: 4.0-8.1)	NR	2 participants
NCT04116658	100	EO2041 (1,2mg) multi-peptide therapeutic vaccine + Nivolumab (anti-PD1) + Bevacizumab (anti-VEGF)	Recurrent Glioblastoma	12.1 months (95% CI: 8.0-14.8)	NR	NR
NCT02455557	66	SurVaxM (surviving peptide cancer vaccine) + TMZ	Newly Diagnosed Glioblastoma	25.8 months (95% CI: 19.5-43.5)	PFS at 6 months; 95% (95% CI: 86-98)	NR
NCT02974621	70	Arm 1: Olaparib (PARP inhibitor) + Cediranib Maleate (VEGFR tyrosine kinase inhibitor)	Recurrent Glioblastoma	316 days (95% CI: 178-459)	118 days (95% CI: 63-172)	NR
		Arm 2: Bevacizumab (anti-VEGF)		218 days (95% CI: 135-368)	92 days (95% CI: 45-142)	
NCT02743078	3	Bevacizumab (anti-VEGF) + Tumor Treating Fields	Bevacizumab Refractory Recurrent Glioblastoma	OS at 21.8 months; 1 participant	PFS at 21.8 months; 0 participants	NR
NCT02337686	18	Pembrolizumab (anti-PD-1) + Surgery	Recurrent Glioblastoma	20 months (95% CI: 8.64-28.45)	PFS at 6 months; 15 participants	3 participants with response
NCT01814813	90	HSPPC-96 (heat shock protein peptide complex-96 vaccine) + Concomitant Bevacizumab (anti-VEGF)	Recurrent Glioblastoma	6.6 months (95% CI: 5.4-10.4)	3.7 months (95% CI: 2.9-5.4)	NR
NCT01609790	137	Bevacizumab (anti-VEGF) + AMG 386 (angiopoietin-1/2 inhibitor)	Recurrent Brain Tumors	7.5 months (95% CI: 6.8-10.1)	4.2 months (95% CI: 3.7-5.6)	Radiographic Response Rate; 4.2% (95% CI: 0-9.8)
NCT04396860	159	Ipilimumab (anti-CTLA-4) + Nivolumab (anti-PD1) + RT	Newly Diagnosed MGMT Unmethylated Glioblastoma	NR	7.7 months (95% CI: 6.5-8.5)	NR
NCT01648348	116	Bevacizumab (anti-VEGF) + TRC105 (anti-endoglin)	Recurrent Glioblastoma	7.4 months (95% CI: 6.5-12.7)	3.2 months (95% CI: 2.6-4.6)	NR
NCT01730950	182	Bevacizumab (anti-VEGF) + RT	Recurrent Glioblastoma	OS at maximum follow up; 21.6% (95% CI: 12.1-31.1)	PFS at 6 months; 54.3% (95% CI: 42.9-65.4)	29.9% (95% CI: 20.0-41.4) with response
NCT02327078	307 (n=33 GBM)	Epacadostat (ID01 inhibitor) + Nivolumab (anti-PD1)	Solid Tumors	OS at 9 months; 45.8% (95% CI: 29.7-60.5)	1.84 months (95% CI: 1.78-2.63)	NR
NCT02667587	716	Nivolumab (anti-PD1) + RT + TMZ	Newly Diagnosed Glioblastoma	OS at 12 months; 82.7% (95% CI: 78.3-86.3)	10.64 months (95% CI: 8.90-11.79)	NR
NCT02617589	560	Nivolumab (anti-PD1) + RT	Newly Diagnosed Glioblastoma	13.40 months (95% CI: 12.62-14.29)	6.01 months (95% CI: 5.56-6.21)	NR
NCT01740258	68	Bevacizumab (anti-VEGF) + RT + TMZ	Newly Diagnosed Glioblastoma	17.8 months (95% CI: 15.2-23.2)	9.9 months (95% CI: 8.8-13.6)	NR
NCT03718767	62	Nivolumab (anti-PD1) for Hypermutated IDH mutant gliomas	IDH-mutant gliomas	7.4 months (95% CI: 4.7-NA)	PFS at 6 months; 25% (95% CI: 7.5-83.0)	NR
		Nivolumab (anti-PD1) for Non-hypermutated IDH mutant gliomas		31.6 months (95% CI: 10.2-NA)	PFS at 6 months; 40% (95% CI: 25.8-62.0)	

## Immune Checkpoint Inhibitors

### Preclinical investigation

Over the past decade, pre-clinical research has rapidly diversified immune-checkpoint strategies for GBM. For many tumors, anti-PD-1 monotherapy has successfully programmed T cells to anti-tumor active states; however, upon ICI administration GBM exhibits adaptive strategies to express other checkpoint activators. As ICI monotherapy did not result in survival benefit for GBM in Phase III trials, current work is developing ICI in combination therapy with both other biologic immunotherapies and standard GBM therapies (e.g.: chemoradiation). Early demonstration of anti-PD-1 efficacy in murine GBM models showed that anti-PD-1 monotherapy improved survival and that there was a synergistic effect when combined with stereotactic radiosurgery (SRS) in overall survival (control: 25 days, anti-PD-1: 27 days, SRS: 28 days, anti-PD-1+SRS: 53 days) (37). Dual therapy with SRS improved survival in another study when combined with anti-PD-1 and anti-TIM-3, with triple therapy having the highest overall survival in the murine GBM model (38). Phase I trial results in recurrent GBM patients of combining hypofractionated stereotactic re-irradiation with anti-PD-1, anti-CTLA-4, anti-VEGF resulted in overall survival of 15.6 months and PFS of 7.1 months, with an acceptable safety profile (39). Local chemotherapy is another important combination candidate with ICI and immunotherapy biologics. Preclinical murine studies identified that local chemotherapy enhances anti-PD-1 efficacy while systemic chemotherapy has the opposite effect (40). While systemic chemotherapy combination resulted in systemic and tumoral lymphodepletion and concordantly decreased immune memory, local chemotherapy increased dendritic cell infiltration into the tumor. This is especially important as trials for combination of anti-PD-1 with TMZ in GBM that found no survival benefit with the combination were done using systemic TMZ therapy (41). These preclinical and early clinical results evidence the need to study the combination of ICI and other immunotherapy biologics with SRS and local chemotherapy in larger trials.

Beyond SRS and local chemotherapy, alternative ICI targets have been characterized. In addition to PD-1 and CTLA-4, V-domain Ig suppressor of T cell activation (VISTA) has emerged as a relevant checkpoint in GBM. It is highly expressed on tumor-associated myeloid cells and contributes to T cell dysfunction via reduced TLR downstream signaling and augments MDSC immunosuppression (42). Preclinical studies suggest that VISTA blockade may relieve myeloid-mediated suppression and enhance effector T cell responses, demonstrating improved T cell infiltration and activation, supporting its inclusion in combinatorial checkpoint strategies.

The combination of ICI with therapies aimed at adaptive and innate ICI resistance, largely driven by immunosuppressive TAMs is the focus of recent preclinical work. As illustrated in **Figure 1** (right panel), checkpoint inhibitors and myeloid-targeted antibodies converge on T cell and APC interactions within the tumor microenvironment. IL-10 expression has been increasingly implicated as an adaptive resistance mechanism to ICI in GBM. Oxidative-stress–driven microglial IL-10 skews macrophages to contribute to immune exhaustion via IL-10 and CD206 upregulation (43), a profile that directly correlates with elevated PD-1/TIM-3 expression and functional exhaustion of infiltrating CD8^+^ lymphocytes (44). In mice, microglial inhibition with anti-PD-1 doubled median survival relative to either monotherapy. IL-10 signaling is a druggable target to relieve T-cell exhaustion in GBM (45). Additionally, IL-6 is a major regulator of myeloid derived suppressor cell (MDSC) formation in GBM, thus serving as a key regulating contributor to the overall immunosuppressive tumor microenvironment (46–49). Anti-IL-6 therapy combined with an anti-CD40 antibody which restored anti-tumor myeloid activation, reversed immunosuppression, and when layered onto PD-1 + CTLA-4 blockade, drove complete tumor regression in a murine model of glioma (50). Myeloid reprogramming via cytokine inhibition is a promising combination therapy with other ICIs. Multiple other TAM targeting strategies include EGFR targeted immunotoxins that establishing durable, tumor-specific memory (51) and the colony-stimulating-factor-1 receptor (CSF-1R) axis which is known to induce conversion of microglia and infiltrating peripheral monocytes into pro-tumorigenic immune cells (52). However, prolonged CSF-1R therapy has been demonstrated to elicit a compensatory TGF-β–driven fibroblast scar perivascularly, encase residual tumor cells and dampen antitumor immunity, ultimately fostering lethal recurrence (32). Co-targeting TGF-β or fibrosis-associated pathways abrogates scar formation and suppresses tumor recurrence (32). This delicate balance in targeting myeloid axes may be a good fit for antisense oligonucleotides. Antisense oligonucleotides represent a potential therapeutic approach that offer the ability to suppress synthesis intracellularly and may avoid BBB permeability challenges as well as potentially reduce some adaptive mechanisms (53). Future studies should investigate the potential synergy of combining these myeloid reprogramming strategies with ICI.

### Clinical trials

A proof-of-concept neoadjuvant case showed that a single dose of triple checkpoint blockade—nivolumab (anti-PD-1), relatlimab (anti-LAG-3) and ipilimumab (anti-CTLA-4)—administered 12 days before resection markedly increased intratumoral CD4^+^ and CD8^+^ T-cell density, activation and clonotype diversity; nivolumab was detected on tumor-infiltrating lymphocytes (TILs), confirming systemic antibodies can reach the brain. Seventeen months later the patient remained recurrence-free, prompting formal evaluation in the ongoing GIANT trial (NCT06816927) (54). In addition, multiple human trials are exploring different ICI combinations with other classical treatment strategies. NCT04225039 couples stereotactic radiosurgery with INCMGA00012 (anti-PD-1) plus the GITR agonist INCAGN01876 to “heat up” recurrent disease. NCT02337491 showed that adding bevacizumab to pembrolizumab was safe but did not improve outcomes over historical bevacizumab controls, underlining the limits of angiogenesis–checkpoint combinations and setting benchmarks for future additive regimens. Finally, NCT02336165 profiles durvalumab monotherapy across five GBM cohorts, generating biomarker-driven selection criteria. While initial studies of anti-PD-1 for newly diagnosed and recurrent GBM did show few responders to therapy, there was a lack of biomarkers. By subtyping GBM, future clinical trials can potentially concentrate a patient cohort that significantly responds to ICI.

While much effort has focused on reversing myeloid-driven immunosuppression and restoring adaptive T cell function, emerging data suggest that immune escape in GBM is not solely governed by classical checkpoint or cytokine networks. In addition, glioblastoma co-opts neural circuitry itself as a pro-tumorigenic signaling axis, linking synaptic integration and activity-dependent signaling to tumor growth and immune resistance. Thus, targeting neuron–glioma crosstalk represents a complementary biologic strategy aimed at disrupting non-immune drivers of tumor persistence that may indirectly reinforce immunosuppressive states.

### Targeting synaptogenesis and axonogenesis in GBM

Recent studies have characterized GBM as a malignancy that is electrically and synaptically integrated within the neural circuitry of the brain (55–58). More specifically, GBM cells form α-amino-3-hydroxy-5-methyl-4-isoxazole-propionic-acid (AMPA)–receptor-dependent excitatory synapses with neighboring neurons and participate in activity-evoked extracellular-potassium currents (55, 56). These inputs depolarize tumor membranes, accelerate proliferation and invasion, and can be attenuated by genetic disruption of AMPA receptors or pharmacological blockade of AMPA with Perampanel, thereby prolonging survival in murine models (55, 56). Extending this paradigm, a study by Taylor and colleagues demonstrated that neuron-to-glioma synapses undergo activity-regulated plasticity through the following mechanism: brain-derived neurotrophic factor (BDNF) acting through TrkB–CaMKII signaling increases AMPA-receptor trafficking, amplifies depolarizing currents and drives tumor growth (58). Genetic or pharmacological inhibition of TrkB arrests progression and improves survival in pediatric and adult xenografts (58). Complementing these mechanistic insights, intra-operative electrophysiology and site-directed biopsies from patients revealed that GBM remodels large-scale language circuits (57). Specifically, regions of high tumor–brain functional connectivity are enriched for synaptogenic, thrombospondin-1–secreting tumor subpopulations, and the extent of this connectivity correlates with poorer cognition and reduced overall survival—effects that can be mitigated by gabapentin-mediated thrombospondin-1 blockade (57). Together, these findings position neuron–glioma electrochemical crosstalk, its BDNF-driven plasticity and circuit-level remodeling as convergent drivers of GBM pathogenesis and highlight anti-synaptogenic strategies as rational therapeutic avenues.

From a therapeutic standpoint, Venkatesh et al. showed that genetic ablation of NLGN3, or systemic inhibition of its sheddase ADAM10 (INCB7839), arrests orthotopic glioma xenografts and prolongs mouse survival—establishing NLGN3 as a druggable extracellular dependency rather than just a biomarker (59). While no neutralizing antibody has yet entered the clinic, humanized anti-NLGN3 IgGs and bispecifics could serve as next-generation “synapse-checkpoint” inhibitors that could replace or synergize with traditional ICIs. Additionally, EphA2 and EphA3, originally defined as axon-path-finding receptors, are up-regulated in GSCs, tumor microtubes and perivascular niches (60). A pre-clinical study with antibody–drug conjugates (ADI-1C1 for EphA2, PAY-loaded huE19 for EphA3) or anti-EphA3 CAR-T cells eliminate patient-derived organoids and extend survival in multiple murine GBM models (60). Neuropilin-1 (NRP1) is a glycoprotein that was found to be a contributor of glioma invasiveness (61). When NRP1 was silenced, either through siRNA inhibition or CRISPR-mediated knockout, there was a down-regulated surface β3-integrin (ITGB3), producing an anti-invasive effect (61). Additionally, NRP1 signaling increases TGF-β; therefore, future work may combine this approach with other immunotherapeutic modalities to curb the infiltration of GBM as well as its immunosuppressive effects (61).

Although targeting neuron–glioma synaptic integration addresses a distinct axis of tumor progression, it does not directly modulate the soluble signaling networks that coordinate immune cell recruitment and polarization within the tumor microenvironment. Consequently, parallel efforts have focused on cytokine and immunomodulatory protein engineering to reshape these communication pathways and restore immune effector function in GBM.

## Cytokines and immunomodulatory proteins

Given the central role of cytokines in cellular communication, immunomodulation, and tumorigenesis, harnessing biologics to modulate these signals represents a promising therapeutic strategy. However, because cytokines are inherently unstable and can exert broad, nonspecific effects, it is critical that their use be precisely tailored to the tumor context (62). To this end, multiple preclinical and clinical studies have investigated cytokines and other immunomodulatory proteins as potential therapies for GBM. The cytokine strategies depicted in **Figure 1** (upper left) are designed to reshape soluble signaling networks that regulate myeloid polarization and T cell activation within the GBM microenvironment.

### Preclinical investigations

Cytokines and immunomodulatory proteins for GBM therapy are under development in preclinical research. Modulation of CD4^+^ and CD8^+^ T cells with NT-I7, a long-acting IL-7, has been explored. IL-7 plays a critical role in T cell maturation and homeostasis. In GL261 glioma-bearing mice, treatment with NT-I7 combined with radiation, or with radiation plus TMZ significantly prolonged survival. While NT-I7 alone did not improve survival, its potential as an adjuvant to standard therapies remains promising (63). Another promising approach involves targeting IL-13 receptor alpha 2 (IL13Rα2) and ephrin type A receptor 2 (EPHA2), which are receptors expressed in GBM and absent within healthy brain tissue. The Pseudomonas exotoxin A (64), was bound to ephrin A1 (eA1) and IL-13 resulting in median PFS of 187 days, a median tumor reduction of 42%, and reduced tumor volumes in 15/16 canines at 42 days following treatment (65). Another approach involves fusing the monoclonal antibody L19 with cytokines such as tumor necrosis factor (TNF), IL-12, and IL-2, generating the fusion proteins L19-TNF, L19-IL12, and L19-IL2. L19 targets the alternatively spliced extra-domain B of fibronectin, a site highly expressed in GBM, enabling selective delivery of the fused cytokines. In murine models, these antibody–cytokine conjugates cross the BBB and localize to syngeneic tumors. Treatment with L19-TNF and L19-IL12 significantly prolonged survival in GL261 glioma-bearing mice, whereas L19-IL2 conferred little to no survival benefit. L19-IL12 enhanced infiltration of CD4^+^ and CD8^+^ T cells as well as natural killer (NK) cells, whereas L19-TNF primarily increased NK cell infiltration. In contrast, L19-IL2 elicited both proinflammatory and immunosuppressive responses (66). Due to its efficacy, L19TNF was later tested in combination with the chemotherapy agent chloroethyl-cyclohexyl-nitrosourea (CCNU). The combination outperformed L19-TNF monotherapy in murine models of GL261 and CT-2A gliomas. Early-phase clinical evaluation (NCT04573192) yielded encouraging results, with 2 of 6 patients demonstrating strong responses (67). These findings suggest that leveraging L19 as a GBM-homing signal to modulate cytokine activity may meaningfully impact disease progression.

Delivery mechanism for cytokine and immunomodulatory protein therapies is important for limiting toxicity and adverse effects in addition to limiting systemic immune effects, given the complex landscape of peripheral immunosuppression (68, 69). Cells can be engineered to deliver cytokines directly to tumors. In CT-2A glioma-bearing mice, tumor resection followed by treatment with inactivated tumor cells engineered to secrete interferon-β (IFN-β) and GM-CSF led to significantly prolonged survival and robust immune cell infiltration within the tumor (70). Also, CAR-T cells engineered to express IFN-α2 and IL-12 demonstrated superior antitumor activity in glioma models compared to conventional CAR-T cells (71). Similarly, hematopoietic stem cells engineered to deliver IFN-α and IL-12 directly to gliomas achieved more effective cytokine delivery than systemic administration and resulted in robust tumor inhibition (72). Other cell-based delivery mechanisms have been developed. TNF-related apoptosis-inducing ligand (TRAIL), which induces apoptosis by binding death receptors 4 and 5, both highly expressed in GBM, has been preclinically investigated as a GBM therapy. To facilitate delivery across the BBB, tumor localizing mesenchymal stem cells were engineered to secrete TRAIL. These engineered cells were also modified to also secrete FMS-like tyrosine kinase 3 ligand (FLT3L), which promotes the maturation of antigen-presenting dendritic cells (DCs). In CT-2A glioma–bearing mice, TRAIL delivery increased survival but failed to reduce tumor volume or elicit a robust T cell response. Co-secretion of FLT3L did not substantially improve outcomes. However, TRAIL combined with FLT3L secretion following tumor resection significantly prolonged survival, decreased recurrent tumor volume, and increased tumor infiltrating CD8+ T cells, highlighting its potential as an adjunct to surgical resection (73). Collectively, these findings highlight the potential for cell-based approaches as platforms for localized cytokine therapy in GBM.

Viral vectors have also been explored as platforms for delivering immunomodulatory biologics. A retroviral replicating vector encoding interferon regulatory factor 8 (IRF8), a key transcription factor for the type 1 conventional dendritic cell (cDC1) lineage, was used in murine glioma models. This approach reduced tumor volume, prolonged survival, and enhanced intratumoral infiltration of cDC1 and CD8^+^ T cells (74). Another study investigated the viral delivery of C-C motif ligand 5 (CCL5) by targeting epidermal growth factor receptor (EGFR). This treatment also improved survival and increased activated immune cell infiltration into the CT-2A glioma bearing mice (75).

### Clinical trials

A non-randomized phase 2 trial (NCT04013672) evaluated the combination of pembrolizumab and SurVaxM, a cancer vaccine, in patients with recurrent GBM who had failed chemotherapy and radiation and had not received immunotherapy. While the primary focus of the trial was to assess pembrolizumab and SurVaxM, Sargramostim was also administered as part of the regime (76, 77). Sargramonstim, also known as recombinant human granulocyte-macrophage colony-stimulating factor (rhu GM-CSF), is a myeloid hematopoietic growth factor that promotes a proinflammatory state and has historically been used as a tumor vaccine adjuvant (78). Among the 41 enrolled patients, 34.5 (95% CI:19.8 to 49.6) had progression free survival (PFS) at 6 months from treatment. rhu GM-CSF was used as a vaccine adjuvant in another non-randomized phase 2 trial (NCT03927222). In this study, patients were pre-conditioned with tetanus-diphtheria toxoid following TMZ and radiation therapy. Patients then received Human CMV pp65-LAMP mRNA-pulsed autologous DCs alongside adjuvant GM-CSF. Median overall survival (mOS) for the six patients studied was 17.4 months (95% CI: 13.3–NA) (79). While no direct comparison was performed to evaluate the effect of rhu GM-CSF in either of the two studies, these results suggest its potential role as an immunomodulatory agent (77, 79).

GM-CSF was compared against polyinosinic-polycytidylic acid stabilized with polylysine and carboxymethyl cellulose (Poly-ICLC) in a phase 1/2 clinical trial (NCT02078648). This trial studied the effect the cancer vaccine SL-701, and GM-CSF and Poly-ICLC were used as vaccine adjuvants (80). Poly-ICLC is a double-stranded ribonucleic acid known for eliciting a broad cytokine release and promoting T cell and NK cell activation (81). In the study, one experimental treatment arm received SL-701 with GM-CSF and the other received SL-701 with poly-ICLC followed by bevacizumab. Final results were posted on the clinical trial registry but have not been peer reviewed. Overall survival at 12 months (OS-12) was similar across treatment groups with 43.5% for SL-701 + GM-CSF and 50.0% for SL-701 + Poly-ICLC + bevacizumab. Treatment was also well tolerated between groups with no participants experiencing regime-limiting toxicity or sudden/unexplainable death. However, the SL-701 + Poly-ICLC + bevacizumab cohort experienced improved objective response rate of 14.3 (95% CI: 5.0 to 29.8) versus the SL-701 + GM-CSF cohort that with a rate of 2.2 (95% CI: 0.1 to 9.9). Again, the vaccine adjuvants did not have direct comparisons with this trial, making definitive claims on their efficacy challenging. Also, the role that bevacizumab plays in treatment response is unclear since it had no comparison either (80). However, the use of vaccine adjuvants alongside cancer vaccines remains an option for treatments seeking to optimize immunomodulation.

Similar efforts have attempted to develop cytokines for combination with adoptive cell therapies. A non-randomized phase 2 trial (NCT01454596) evaluated EGFRvIII-targeted CAR-T cells in combination with chemotherapy, with aldesleukin administered as an adjuvant across all treatment arms (82). Aldesleukin, an IL-2 historically used to treat metastatic melanoma (83), was included to support T cell activity. The primary aim of the study was to assess the safety of EGFRvIII-directed CAR-T cells, however, the therapy did not result in meaningful survival benefit or tumor response (84). In the absence of a control group, the specific contribution of IL-2 to the immune response remains unclear, raising questions about the utility of cytokine adjuvants in future CAR-T cell strategies.

In contrast to vaccine and adoptive cell therapy adjuvants, MDNA55 takes a targeted cytotoxic strategy. This biologic was produced by fusing IL-4 to a modified Pseudomonas aeruginosa exotoxin A (PE). The IL-4 receptor (IL4R) is widely expressed in both GBM cells and immunosuppressive myeloid derived suppressor cells with the TME. MDNA55 binds to IL4R, is endocytosed, and then delivers the toxic PE payload to induce apoptosis (85). A phase 2 non-randomized trial (NCT02858895) used Convection-Enhanced Delivery (CED) to deliver the drug past the blood brain barrier (BBB). Within the intent-to-treat (ITT) population, the mOS of the 47 patients was 10.2 (one-sided 80% CI: 8.39 to 12.75), and OS-12 was 43% (95% CI: 29%–57%). For the per-protocol (PP) population, the mOS of the 44 patients was 11.64 (one-sided 80% CI: 8.62 to 15.02), and OS-12 was 46% (95% CI: 31%–60%). Treatment was well-tolerated by all but two patients, and most adverse events were neurological due to GBM disease burden (85, 86). Given these results and its favorable safety profile, MDNA55 represents a potential therapeutic strategy for GBM, leveraging IL-4–mediated targeting to deliver a cytotoxic exotoxin selectively to tumor cells.

Despite some progress, several challenges remain before cytokines and related immunomodulatory proteins can be effectively leveraged to treat GBM. One major hurdle is the translation of findings from animal models to humans. Even subtle differences in receptor expression or timing can have dramatic consequences for translating highly targeted interventions (73). Furthermore, immune signaling molecules often display dual and context-dependent roles. For example, TRAIL has been shown to both inhibit tumor growth and support T cell activity in one setting, while inducing T cell apoptosis in another (73, 87). Adding to the complexity, immune signaling networks are highly redundant (88), meaning that targeting a single cytokine may be insufficient to achieve durable therapeutic effects. This points to the need to study complex combination therapies (63). However, the vast number of possible cytokine combinations, coupled with the practical limitations of tumor models, presents a significant barrier to exhaustively testing these strategies and suggesting a role for high throughput techniques or machine learning (65). Perhaps the greatest hurdle is that systemic administration of cytokines is not feasible due to their toxicity and severe side effects (66, 89). Effective application will therefore require delivery strategies that are tumor-specific and restrict distribution to healthy tissues. While engineering cells to secrete cytokines in a targeted manner offers one solution, this approach is unlikely to be practical as a primary treatment strategy but does offer a combination therapy opportunity with adoptive cell therapies (70).

## Adoptive cell therapies for GBM

### Clinical trials of CAR-T cells

To date, multiple early-phase clinical trials have investigated chimeric antigen receptor (CAR) T cells, T cells engineered to recognize and target specific GBM neoantigens, with limited clinical efficacy. As shown in **Figure 1** (lower left), adoptive cell platforms directly target tumor-associated antigens while variably modulating the surrounding immune microenvironment. One of the first targets was the tumor-specific Epidermal Growth Factor Receptor Variant III (EGFRvIII), expressed in roughly 30% of GBMs (90). In 2019, a phase I dose escalation trial of third-generation EGFRvIII-directed CAR-T cells in recurrent GBM (NCT01454596) following lymphodepleting chemotherapy was tolerated at lower dose levels, only reaching dose-limiting pulmonary toxicity at the study’s highest concentration of ≥10 (10) CD3+ cells (91). However, the treatment did not produce any objective tumor or radiographic responses, and 16 of 17 evaluable patients progressed within 3 months. Subsequently in 2024, one trial of newly diagnosed EGFRvIII+ GBM patients repeated peripheral infusions of the CAR-T combined with pembrolizumab (92). Once again, the treatment was safe but had limited clinical efficacy. Of note, paired tumor analyses demonstrated increased T-cell exhaustion and regulatory programs at relapse. Together, these studies demonstrate that innate tumor heterogeneity, antigen loss, and TME-driven adaptive immunosuppression are potential resistance mechanisms by which GBM may abrogate EGFRvIII CAR-T activity.

Redirecting CARs to alternative antigens, such as HER2 and IL13Rα2, has similarly encountered innate and adaptive barriers. Human epidermal growth factor receptor 2 (HER2) is overexpressed in up to 80% of glioblastomas (93), making it an attractive antigen for targeted adoptive cell therapies. IL-13 receptor alpha 2 (IL13Rα2), a membrane receptor of IL-13 is overexpressed on around half of all GBMs (94). In one pilot study, three patients received multiple intracavitary infusions of IL13Rα2 CAR-T cells via indwelling catheters placed in the resection cavity, which was well tolerated and led to transient regression of intracranial and spinal tumors, with CAR-T cells detected in the cerebrospinal fluid indicating trafficking throughout the CNS (95). Building upon this, a 2024 phase I trial of 65 patients with recurrent high-grade glioma showed that locoregional infusion of IL13Rα2 CAR-T cells was both feasible and safe, with no dose-limiting toxicities and evidence of clinical activity (96). Half of the patients achieved stable disease or better, and a small number experienced radiographic partial or complete responses. The most favorable outcomes were seen in the cohort receiving dual intratumoral and intraventricular infusions with memory-enriched CAR-T products, where median overall survival in the recurrent GBM subgroup reached 10.2 months compared with 7.7 months across the entire study population. Still, objective radiographic responses were rare (3 of 58 evaluable patients), most patients experienced only transient disease stabilization, and outcomes were strongly influenced by pretreatment intratumoral T cell infiltration. Moreover, IL13Rα2 expression was heterogeneous across tumors, highlighting the risk of antigen escape.

### Resistance mechanisms and next-generation strategies

As evidenced by the aforementioned clinical trials, GBM presents an immunosuppressive milieu which abrogates the efficacy of various adoptive cell therapies. Although most clinical trials have shown that adoptive cell therapies are safe and efficacious, next generation therapies must improve upon the clinical efficacy of these treatments. Of the many innate and adaptive resistance mechanisms which GBM presents, one significant challenge is antigenic heterogeneity. Unlike B-cell leukemias which uniformly express CD19, GBM tumors are notoriously heterogeneous, often containing mixtures of antigen positive and negative cells. This allows for immune escape by antigen loss, whereby the expression of a single antigen may be downregulated if it is the sole target of an adoptive cell therapy, which was demonstrated by the EGFRvIII CAR-T trial (97). Even when CAR-T cells effectively killed EGFRvIII-expressing glioma cells, the remaining tumor simply consisted of EGFRvIII-negative cells, leading to relapse. Similarly, other targets like HER2 and IL13Rα2 are not expressed on every cell in a tumor, and heterogeneous expression means that a single-antigen CAR-T therapy can leave some cells untouched. One strategy to account for the intratumoral heterogeneity of GBM neoanitgens is the SynNotch-CAR-T. These CAR-T cells are only active upon being locally induced by recognizing their target antigen. This strategy prevents off-tumor killing, T cell anergy, and the need for homogenous expression of the target antigen on tumor cells. Preclinical studies in murine models demonstrated increased antitumor efficacy and durability of the constructs compared to conventional CAR-T cells (98). The SynNotch safety mechanism is a powerful tool that can be combined with other antigen targeting. Whereas EGFRvIII is very specific to GBM but not highly homogenously expressed across the tumor, there are other antigens including EphA2 and IL-13Rα2 that are less specific for GBM but more highly expressed across the tumor. A Phase I study for newly diagnosed and recurrent EGFRvIII+ GBM is trialing anti-EGFRvIII SynNotch CAR-T cells that upon activation are induced to also target EphA2 and IL-13Rα2.

One limitation of CAR-T cells is that they do not increase the cytotoxicity or targeting of other local T cells and therefore do not exhibit response amplification while requiring large numbers of the constructs which are susceptible to anergy. The lack of professional APC and CD4+ priming results in limited endogenous response or epitope spreading. To address GBM’s intratumoral heterogeneity and the lack of endogenous T cell amplification, CAR-T cells with a T-cell-engaging-molecule (TEAM) have been developed. CARv3-TEAM-E T cells, in addition to specific targeting of EGFRvIII tumor cells, upon activation secrete a bispecific antibody that tethers EGFRwt tumor cells to CD3 T cells including the CAR-T construct or bystander T cells in the TME. Phase 1 study in only three patients of this construct in human recurrent GBM patients demonstrated rapid radiographic tumor regression within days of a single intraventricular infusion (99). However, eventual tumor progression occurred in two of three patients demonstrating the complex adaptive response to the therapy and potential for synergistic immune therapies to enhance long term memory and anti-tumor activity. Another next-generation strategy to prevent antigen escape is to target multiple antigens simultaneously. Tandem CAR-T constructs co-targeting multiple GBM-associated antigens (EGFRvIII, IL-13Rα2) demonstrated a long-term, complete, and durable response which was not achieved by comparison arm of single-antigen CAR-T cells in preclinical models (100). Bivalent CAR-T cells, which co-target two tumor antigens, aim to kill heterogeneous tumor cell populations and reduce the chance of immune evasion. In a recent phase I UPenn trial of dual EGFRvIII/IL13Rα2 CAR-T cells, all six treated recurrent GBM patients showed early MRI reductions in tumor burden however these findings represent early interim data from a small cohort. While Modified Response Assessment in Neuro-Oncology (mRANO) objective responses were not yet achieved in this small cohort, these findings strongly motivate broader testing of dual- and multi-antigen CAR constructs to determine whether they can outperform monovalent approaches (101). One current trial, BrainChild-04, is even investigating a quad-CAR-T construct for treating DIPG, DMG, and other CNS tumors by simultaneously targeting B7-H3, EGFR, HER2, and IL13Rα2 (102). Of note, this is a first in human early phase trial without mature efficacy data at this time. One major benefit of targeting multiple antigens is that it allows to not only target the tumor but also to target immunosuppressive axis around the tumor. In parallel, tenascin-C–specific CAR-T cells targeting a tumor-enriched extracellular matrix glycoprotein showed selective tumor cell killing with significant murine survival benefit, supporting the feasibility of ECM-directed CAR strategies (103).

In all, CAR-T cell trials in GBM have demonstrated that locoregional delivery is safe and biologically active, but durable responses remain rare, limited by antigen heterogeneity, adaptive antigen loss, and profound immunosuppression in the tumor microenvironment. These shortcomings have motivated both preclinical innovations to enhance CAR function as well as parallel exploration of other adoptive T cell strategies. While logic-gated CAR-T systems address antigen heterogeneity and spatial specificity, their efficacy remains constrained by T cell exhaustion and the suppressive glioblastoma microenvironment. This has prompted exploration of alternative innate-like lymphocyte platforms, including NK, NKT, and γδ T cells, which may offer complementary persistence, reduced alloreactivity, and distinct mechanisms of tumor recognition.

### Other adoptive cell transfer approaches

Beyond CAR-T cells, several other adoptive cell strategies have been explored in GBM. Adoptive NK cell therapies have been built to enhance innate anti-tumor immune activity. In GBM, IL-21 expressing NK cells via CEBPD are reprogrammed to have enhanced long-term cytotoxicity and metabolic fitness (104). Recent targeting of GBM with NK cells in a similar approach to CAR-T cells has led to the development of CAR-NK cells. In nine patients with recurrent glioblastoma, intracranial injection of CAR-NK cells using a HER2 chimeric antigen was found to be feasible and safe with injections ranging from 7 to 37 weeks and no patients developing cytokine release syndrome (105). Preliminary efficacy results include five of nine patients showing stable disease following relapse surgery. In recurrent GBM human patients, a Phase 2 trial (NCT06061809) of combination of CAR-NK cell therapy (PD-L1 target), IL-15 agonist, and tumor treating fields (TTFs) released preliminary results of 12 patients (3 of which received TTFs) who received 57 total doses, with one patient at the time with ongoing complete radiographic response and resolved lymphopenia where eight other patients demonstrating increased and maintained lymphocyte count from baseline (106). Of note, these results are preliminary and not peer reviewed. Recent work has applied synNotch engineering to iPSC-derived NK cells to reprogram immunosuppressive signaling in glioblastoma (107). By converting the inhibitory TIGIT–CD155 interaction into an activating synNotch signal that induces CD73 blockade, this platform simultaneously disrupts checkpoint and adenosine-mediated immunosuppression. In orthotopic patient-derived GBM models, these engineered NK cells enhanced cytotoxicity, reprogrammed the tumor microenvironment, and achieved complete tumor eradication, supporting their potential as an allogeneic, programmable adoptive therapy.

CAR macrophages have similarly been constructed and trialed in glioma models, and while CAR T, NK, and macrophages have distinct effects on the GBM TME, their combination with cytokines demonstrates a similar improved effect (108). Adoptive CAR therapies are a promising intervention that should be further investigated and combined with other immunotherapies including other adoptive T cell therapies and cytokine therapies.

Beyond conventional CAR-T and NK platforms, recent work has expanded adoptive strategies to include innate-like T cell subsets. Allogeneic CAR-NKT cells have demonstrated preclinical activity in glioblastoma models, leveraging their inherent ability to recognize CD1d-presented glycolipids while simultaneously delivering CAR-mediated cytotoxicity, offering the potential for reduced graft-versus-host risk and off-the-shelf scalability (109). Similarly, engineered γδ T cells represent a promising approach given their MHC-independent tumor recognition and capacity to function within immunosuppressive microenvironments, with recent studies demonstrating feasibility and antitumor activity in GBM-relevant systems (110). Together, these platforms broaden the adoptive immunotherapy landscape by integrating innate immune recognition with engineered specificity, potentially mitigating antigen escape and HLA restriction limitations inherent to conventional CAR-T therapies.

### Preclinical advances

When CAR-T cells infiltrate GBM, they immediately encounter the complex array of GBM immunosuppression (111). In the EGFRvIII CAR trial, along with antigen loss, the recurrent tumors showed upregulation of PD-L1, IDO1 enzyme, and immunosuppressive cytokines after CAR-T infusion (97). This suggests the tumor can adapt by activating redundant immune checkpoints and metabolic suppression to dampen T cell attack. One strategy to combat this is combining CAR therapies with checkpoint blockade, which for CAR-NK cells combined with anti-PD-1 increased T and NK cell infiltration and resulted in successful treatment of murine orthotopic tumors refractory to CAR-NK cell monotherapy (112). Another strategy is the construction of “armored” CAR-T cells that can modulate the environment or resist exhaustion. One approach is engineering CAR-T cells to secrete blockers of suppressive pathways, such as a recent preclinical study which investigated CAR-T cells that constitutively secrete a modified SIRPγ protein, which blocks the CD47 anti-phagocytic signal that tumor cells use to inhibit macrophages (113). In an orthotopic EGFRvIII-mosaic GBM model, these armored CARs enhanced microglia/monocyte-derived macrophage (GAM) phagocytosis and eliminated antigen-negative bystander tumor cells, achieving near-complete clearance. Similarly, CAR-NK cells have been engineered to activate within the GBM TME to suppress the concentration of adenosine via CD73 (114). Other CAR-driven TME reprogramming strategies include CAR-T cells that secrete chemokines to attract additional T cells into the tumor, or those that express dominant-negative TGF-β receptors to render them resistant to TGF-β (115–117). Finally, the lack of truly tumor-specific targets complicates adoptive cell therapy for GBM (118, 119). An ideal target is expressed only on tumor cells and not on essential normal cells. EGFRvIII fits this criterion but is absent in many patients and is not expressed on all tumor cells. An emerging antigen target is advillin, which represents a potentially more tumor-restricted target that may help address the longstanding challenge of on-target/off-tumor toxicity in adoptive cell therapies (120). The identification of an advillin-directed inhibitor further supports its therapeutic tractability and highlights the continued importance of systematic antigen discovery to expand the repertoire of safer, GBM-specific targets.

Current preclinical work and early translational efforts point to several rational avenues to address CAR resistance mechanisms, including multi-antigen and on/off-gated receptors to mitigate antigen escape and improve specificity, armored designs that deliver cytokines or checkpoint/myeloid modulators and incorporate resistance to TGF-β, and approaches to enhance homing and durability. Future trials should integrate prospective tumor and CSF correlative studies, perhaps by utilizing patient derived organoids (121), to track antigen dynamics, T-cell phenotypes, and microenvironmental programs to determine whether next-generation products can produce sustained clinical benefit beyond the transient responses observed to date.

## Reprogramming the ‘cold’ GBM TME: vaccines and oncolytic viruses

While adoptive cell therapy has sought to reprogram the TME by enhanced cytotoxicity, oncolytic viruses and GBM vaccines are two other immunotherapy biologics that attempt to reverse the immunological ‘coldness’ of GBM. One of the most inherent challenges of GBM is that it is a ‘cold’ tumor, with low mutational burden, low cytotoxic immune cell infiltration, and a highly immunosuppressive environment (14). GBM vaccines present selected antigens and prime T cells in lymph nodes to enhance an effector response while addressing the low mutational burden. However, GBM vaccines do not directly change the TME and are dependent on an anti-tumor myeloid environment in the TME to allow for T cell infiltration and cytotoxicity. Furthermore, vaccines rely on functioning dendritic cells for antigen presentation which is often suppressed in GBM. Lastly, the monoclonal priming to the selected antigen risks immune escape. Therefore, vaccines are commonly combined with other immunotherapies (e.g.: poly-ICLC, GM-CSF, STING, ICI) aimed at stimulating an inflammatory response and reversing myeloid driven immunosuppression in the TME. On the other hand, oncolytic viruses are a directly immunogenic therapy that specifically target tumor cells which they infect and lyse, creating a release of antigens and viral pathogen associated molecular patterns (PAMPs) in the TME. This lysis results in pushing the TME to a ‘hot’ state via polyclonal priming, inflammation via Type I Interferon signaling, and the active recruitment of DC, NK, and T cells to the TME. These vaccine and viral platforms are summarized in **Figure 1** (lower right), highlighting their role in priming adaptive immunity and inflaming the otherwise immunologically ‘cold’ GBM microenvironment.

### Vaccines

Therapeutic vaccination in GBM has aimed to generate tumor specific T cell response but clinical efficacy has been limited. Rindopepimut is one experimental vaccine that combines the EGFRvIII antigen to a carrier protein. A phase II clinical trial (ACT III) of Rindopepimut combined with GM-CSF in newly diagnosed EGFRvIII expressing GBM patients demonstrated elevated anti-EGFRvIII antibody titers and elimination of EGFRvIII in four of six tumor samples after three months of therapy (122). However, the phase III randomized clinical trial (ACT IV) in the same patient population compared Rindopepimut and GM-CSF versus control where both groups had concurrent TMZ treatment showed no significant overall survival effect hypothesized to have been limited by antigen heterogeneity and loss (123). There were some preliminary promising phase II results of Rindopepimut with Bevacizumab vs Bevacizumab alone in EGFRvIII expressing recurrent GBM where the vaccine group had significantly increased survival. However, the primary PRS6 endpoint was not significantly increased and only 73 patients were included in the study and thus requires validation in larger sized trials (124). Other vaccine efforts using single antigen design include SurVaxM which targets survivin. Final results demonstrate that the peptide vaccine SurVaxM combined with TMZ and immune adjuvants (Montanide and GM-CSF) in a phase II trial in newly diagnosed GBM patients resulted in mPFS (11.4 months) and mOS (25.9) with PFS6 of 95.2% greater than the estimated 54% rate from historical controls (125). The active multicenter SURVIVE phase II trial aims to evaluate these findings in a larger cohort.

Given the disappointing phase III trial results of single target vaccines, novel vaccine designs have increasingly used autologous tumor lysate to not limit priming to single antigens. DCVax-L is a dendritic cell vaccine that uses autologous tumor lysate pulsed dendritic cells as adoptive therapy following resection of newly diagnosed GBM. A Phase III trial (NCT00045968) compared standard of care with and without DCVax-L following resection of newly diagnosed GBM. A total of 331 patients were included using a crossover design where following recurrence all patients were allowed to receive DCVax-L. The phase III trial found that adding DCVax-L to standard of care significantly increased survival in both newly diagnosed (median OS: 19.3 versus 16.5 months) and recurrent GBM (median OS: 13.2 versus 7.8 months) (126). The success of this trial demonstrates that GBM patients can mount robust peripheral T cell responses. This is further supported by the results of a Phase I trial following resection of newly diagnosed GBM which used patient specific unmutated antigens and predicted neoepitopes identified through individualized genomic, transcriptomic, and immunopeptidomic profiling for vaccination (127). Patients were restricted to those with GBM positive for human leukocyte antigen (HLA)-A*02:01 or HLA-A*24:02. The vaccines that were combined with immune adjuvant poly-ICLC and GM-CSF showed sustained CD8 and CD4 T cell responses against the predicted neoepitopes. However, when patients underwent resection for recurrent tumors, vaccine induced clonotypes were present in blood but barely detectable in tumor specimens, with most T cells localized to perivascular regions and not infiltrating into tumor parenchyma, and an immunosuppressive myeloid TME.

Building on the limitations of prior peptide and dendritic cell–based vaccines, a recently reported personalized mRNA vaccine platform in Glioblastoma has demonstrated encouraging preclinical and early clinical activity (128). In murine models, lipid nanoparticle–formulated mRNA vaccines encoding patient-specific tumor antigens induced rapid dendritic cell activation, type I interferon signaling, and expansion of tumor-reactive T cells, translating into delayed tumor progression. First-in-human data following resection showed the approach is feasible and capable of eliciting rapid systemic and intratumoral T cell responses. Unlike earlier vaccine strategies limited to single epitopes or labor-intensive ex vivo cell platforms, mRNA vaccines enable rapid, multi-antigen personalization with intrinsic innate immune stimulation, offering a scalable strategy to address GBM heterogeneity and immune evasion. However, the poor T cell infiltration of previous trials will likely similarly limit mRNA vaccination paradigms. Taken together these results evidence that while vaccines mount a robust peripheral T cell effector and memory response, the peripheral response is not enough to overcome the GBM TME and necessitates combination with other strategies that can turn the ‘cold’ TME into a ‘hot’ anti-tumor inflammatory environment.

### Oncolytic viruses

Oncolytic viruses (OVs) are among the few agents that can actively inflame GBM’s profoundly ‘cold’ immunosuppressive microenvironment (129) by driving immunogenic tumor cell lysis, inducing type I interferons, and recruiting both innate and adaptive immune effectors. In murine GBM studies, OV therapy armed with IL-12 decreased angiogenesis, decreased Treg in the TME, and increased IFN-γ release (130). The triple combination of an OV expressing IL-12, anti-PD-1, and anti-CTLA-4, cured most mice in two glioma models where following treatment was a macrophage influx of M1-like polarization, distinct from the overwhelming immunosuppressive TAMs that normally characterize the GBM TME (131). The treatment was further associated with an increase in effector vs regulatory T cells. An important result of this study was that CD4 T cells, CD8 T cells, and macrophages were all required for synergistic response, evidencing the power of OVs to reprogram an array of immune cell programs in the GBM TME to anti-tumor roles (131).

In early human trials, OVs have demonstrated some promise, including the OV G47Δ earning approval in Japan for malignant gliomas including GBM (132). A combination OV, targeting tumors by carrying E1A gene deletion rendering the virus incapable of replicating in normal cells with a functional Rb pathway (133), combined with anti-PD-1 ICI in recurrent GBM was tested in a phase I/II trial. The combination of OV and ICI has improved outcomes in melanoma, where responders had increased CD8 T cells, elevated PD-L1, and higher IFN-γ following treatment but response was not correlated with pretreatment CD8 T cell infiltration or IFN-γ signature (134). In recurrent GBM, the combination therapy was found to be both safe as well as have an overall survival at 12 months increased from prespecified controls (OS12: 52.7% versus 20%). Furthermore, patients with objective response had longer survival and three patients with completed treatment had durable responses remaining alive at 45, 48, and 60 months (135). The expression of alternative immune checkpoints (TIGIT, LAG3 and B7-H3) was elevated following treatment in specimens from those with disease progression (135). Further early evidence for the role of OVs in GBM therapy comes from the phase I study of an OV using an engineered herpes virus with viral neurovirulence gene transcribed by a promotor for nestin which is overexpressed in GBM versus healthy differentiated tissue. Results demonstrated safety of the therapy and that the viral serology predicted improved survival. The anti-tumor response was demonstrated in OV seropositive participants by an immune response consisting of peripheral expansion of T cells of multiple clonotypes, increased T cells in tumors, expansion of tissue-resident memory T cell clonotypes, and transcriptomic signatures of tumor immune activation (136, 137). In two patients, CD68+ macrophage populations were present following treatment, specifically in perinecrotic tumor regions (136). Future therapies may seek to develop novel OV designs and combination therapies to increase the inflammatory anti-tumor macrophage response following OV therapy as preclinical studies demonstrated that synergistic effect is dependent on CD4 T cells, CD8 T cells, and macrophages in the TME.

## Discussion

The ‘cold’ immunosuppressive GBM TME has resulted in disappointing performance of most immunotherapy trials. The low mutational burden, large intertumoral and intratumoral heterogeneity, and myeloid derived immunosuppression across multiple axes result in unfavorable responses to mono-immunotherapy. Despite strong preclinical rationale, the translation of biological immunotherapy in GBM has often failed. For example, despite robust response in preclinical models, success in humans across multiple solid tumors, high PD-L1 expression on GBM cells, and evidence of exhausted T cells in the GBM TME, anti-PD-1 therapy has not worked thus far in GBM. Unique factors to the GBM TME have driven this effect including low baseline T cell infiltration, antigen scarcity, non-PD-1 based myeloid immunosuppression, and the trapping of T cells in perivascular spaces. In response to these failed trials, immunotherapy has sought to address these resistance mechanisms, most notably the immunosuppressive myeloid compartment. Anti-PD-1 combined with anti-myeloid CSF-1R inhibition showed strong synergy in murine models (138), but direct myeloid modulation has not shown clinical efficacy in GBM (139). While the murine syngeneic model is an important tool, there is a significant mismatch in antigen availability and immunogenicity between murine models and human GBM. It is likely that human GBM has unique mechanisms of myeloid resistance and immunosuppression. Given the success across multiple therapies in murine GBM, and the disappointing results in human, studying human GBM TME dynamics is paramount. Patient derived organoids, which have demonstrated impressive biomarker ability for response to treatment, represent a promising approach to this problem (121).

The heterogeneous responses observed across immunotherapy trials in glioblastoma suggest that therapeutic efficacy is likely context-dependent and contingent upon the dominant immune and tumor-intrinsic state. While bulk transcriptional subtypes have historically defined GBM as proneural, classical, or mesenchymal, emerging single-cell and spatial analyses indicate that these states coexist within tumors and fail to reliably predict response to immunotherapy. Thus, biologic selection may require stratification based less on static subtype labels and more on functional immune architecture. In this framework, tumors characterized by dense myeloid infiltration and elevated IL-6, TGF-β, or CSF-1R–dependent programs may be preferentially suited to myeloid-reprogramming strategies layered with checkpoint inhibition. Tumors with preserved antigen presentation and detectable tumor-reactive T cell clonotypes, yet limited intratumoral trafficking, may benefit more from inflammatory priming approaches such as oncolytic viruses or cytokine-armed cellular therapies. Similarly, quantitative profiling of antigen density and intratumoral heterogeneity may inform the selection of monovalent versus multi-antigen or logic-gated adoptive platforms to mitigate antigen escape. By aligning dominant immune states with mechanistically matched biologics, future trial design may transition personalized combination of immunotherapies in GBM.

While each biologic platform is often evaluated independently, meaningful clinical responses in GBM will likely require mechanistically rational combinations that address complementary resistance axes. For example, oncolytic viruses may enhance CAR-T efficacy by inducing type I interferon signaling, increasing antigen release, and reprogramming tumor-associated macrophages toward pro-inflammatory states, thereby overcoming myeloid suppression and spatial T cell exclusion. Similarly, myeloid-directed therapies targeting IL-6 or CSF-1R may restore antigen presentation and reduce immunosuppressive cytokine signaling, creating a permissive environment for checkpoint blockade or adoptive cell therapies. Cytokine-armed cellular platforms and logic-gated constructs may further enhance persistence and mitigate antigen escape. Integrating biologics based on dominant, or even personalized, resistance mechanisms will be critical to achieving durable responses in GBM. In parallel, improved understanding of adaptive responses to each therapeutic axis and immunotherapy method will better guide combination selection.

### Future directions

Given the dynamic array of immunosuppressive axes in the GBM TME, the selection of therapies to develop in future combination trials is an extremely difficult task. Detailing the dominant immunosuppressive axis in human GBM over time in response to specific treatment can uncover combination needs. Longitudinal profiling of tumor, CSF, and blood before and during both standard of care and clinical trial therapies can detail immunosuppression and adaptive resistance across myeloid, metabolic, and spatial axes. This may uncover necessary timing components to combination therapies. For example, activating lymphoid cells before myeloid reprograming is complete may accelerate exhaustion. Biomarkers from longitudinal profiling efforts will be crucial to guide sequential combination therapies. Furthermore, combination therapies should include not just the targeting of a single immunosuppressive axis, but also the targeting of its known adaptive response. Patient derived organoids should be included in clinical trials to detail adaptive responses by the myeloid compartment separated by the therapeutic target, to allow future combinations to incorporate targeting of these respective adaptive axes.

Some promising results are noted in recent trials of combination therapies composed of a combination of biologics including ICIs, cytokines, immunomodulatory proteins, CAR-T and CAR-NK adoptive therapies, vaccines, and oncolytic viruses. Across many trials, even those that did not achieve their primary efficacy endpoints, several patients demonstrate long-term response to therapy. Therefore, there is a necessity for improved subtyping of GBM for improved trial design to identify specific therapies and patients with clinical response. Subtyping GBM is a difficult task, what was once thought of as classical subtypes of proneural vs mesenchymal from bulk RNA data has been found to likely be a composite of many co-existing cell states from large snRNA efforts (140). Immune biomarkers from clinical trial results have similarly been limited in separating responders versus non-responders (36, 141). New techniques being used in combination with current clinical trials including serial tumor tissue biopsies and patient derived organoids are promising strategies to improve our biomarkers and subtyping of GBM (121, 137). The development of improved biomarkers and GBM subtyping will guide the appropriate selection and combination of the many promising biologics in GBM immunotherapy to catalyze therapeutic development and improve patient outcomes.

## References

[1] Reck M Rodríguez-Abreu D Robinson AG Hui R Csőszi T Fülöp A . Pembrolizumab versus chemotherapy for PD-L1–positive non–small-cell lung cancer. N Engl J Med. (2016) 375:1823–33. doi: 10.1056/NEJMoa1606774 27718847

[2] Seth R Agarwala SS Messersmith H Alluri KC Ascierto PA Atkins MB . Systemic therapy for melanoma: ASCO guideline update. J Clin Oncol. (2023) 41:4794–820. doi: 10.1200/JCO.23.01136 37579248

[3] Lim M Weller M Idbaih A Steinbach J Finocchiaro G Raval RR . Phase III trial of chemoradiotherapy with temozolomide plus nivolumab or placebo for newly diagnosed glioblastoma with methylated MGMT promoter. Neuro Oncol. (2022) 24:1935–49. doi: 10.1093/neuonc/noac116 35511454 PMC9629431

[4] Reardon DA . Nivolumab monotherapy and nivolumab plus ipilimumab in recurrent glioblastoma: results from CheckMate 143. JAMA Oncol. (2020) 6:7. doi: 10.1001/jamaoncol.2020.1024 32437507 PMC7243167

[5] Omuro A Brandes AA Carpentier AF Idbaih A Reardon DA Cloughesy T . Radiotherapy combined with nivolumab or temozolomide for newly diagnosed glioblastoma with unmethylated MGMT promoter: An international randomized phase III trial. Neuro Oncol. (2023) 25:123–34. doi: 10.1093/neuonc/noac099 35419607 PMC9825306

[6] He L Azizad D Bhat K Ioannidis A Hoffmann CJ Arambula E . Radiation-induced cellular plasticity primes glioblastoma for forskolin-mediated differentiation. Proc Natl Acad Sci. (2025) 122:e2415557122. doi: 10.1073/pnas.2415557122 40009641 PMC11892679

[7] Stupp R Mason WP van den Bent MJ Weller M Fisher B Taphoorn MJB . Radiotherapy plus concomitant and adjuvant temozolomide for glioblastoma. N Engl J Med. (2005) 352:987–96. doi: 10.1056/NEJMoa043330 15758009

[8] Noorani I de la Rosa J . Breaking barriers for glioblastoma with a path to enhanced drug delivery. Nat Commun. (2023) 14:5909. doi: 10.1038/s41467-023-41694-9 37737212 PMC10517119

[9] Seano G Jain RK . Vessel co-option in glioblastoma: emerging insights and opportunities. Angiogenesis. (2020) 23:9–16. doi: 10.1007/s10456-019-09691-z 31679081 PMC7012982

[10] Mikolajewicz N Zhai K Puri A Miletic P Tatari N Wei J . Reactive oligodendrocytes promote glioblastoma progression through CCL5/CCR5-mediated glioma stem cell maintenance. Neuron. (2026) 114:237–249.e10. doi: 10.1016/j.neuron.2025.12.012 41570802

[11] Kang S Ughetta ME Zhang JY Marallano VJ Sattiraju A Hannah T . Glioblastoma shift from bulk to infiltrative growth is guided by plexin-B2-mediated microglia alignment in invasive niches. Nat Cancer. (2025) 6:1505–23. doi: 10.1038/s43018-025-00985-4 40442367 PMC12353550

[12] Khan F Pang L Dunterman M Lesniak MS Heimberger AB Chen P . Macrophages and microglia in glioblastoma: heterogeneity, plasticity, and therapy. J Clin Invest. (2023) 133:e163446. doi: 10.1172/JCI163446 36594466 PMC9797335

[13] Jansen JA Omuro A Lucca LE . T cell dysfunction in glioblastoma: a barrier and an opportunity for the development of successful immunotherapies. Curr Opin Neurol. (2021) 34:827–33. doi: 10.1097/WCO.0000000000000988 34569985 PMC8595795

[14] Jackson CM Choi J Lim M . Mechanisms of immunotherapy resistance: lessons from glioblastoma. Nat Immunol. (2019) 20:1100–9. doi: 10.1038/s41590-019-0433-y 31358997

[15] Liu Y Zhou F Ali H Lathia JD Chen P . Immunotherapy for glioblastoma: current state, challenges, and future perspectives. Cell Mol Immunol. (2024) 21:1354–75. doi: 10.1038/s41423-024-01226-x 39406966 PMC11607068

[16] Li M Sun P Tu B Deng G Li D He W . Hypoxia conduces the glioma progression by inducing M2 macrophage polarization via elevating TNFSF9 level in a histone-lactylation-dependent manner. Am J Physiol Cell Physiol. (2024) 327:C487–504. doi: 10.1152/ajpcell.00124.2024 39010835

[17] Jin X Zhang N Yan T Wei J Hao L Sun C . Lactate-mediated metabolic reprogramming of tumor-associated macrophages: implications for tumor progression and therapeutic potential. Front Immunol. (2025) 16:1573039. doi: 10.3389/fimmu.2025.1573039 40433363 PMC12106438

[18] Jackson C Cherry C Bom S Dykema AG Wang R Thompson E . Distinct myeloid-derived suppressor cell populations in human glioblastoma. Science. (2025) 387:eabm5214. doi: 10.1126/science.abm5214 39818911 PMC12836367

[19] Khan F Lin Y Ali H Pang L Dunterman M Hsu WH . Lactate dehydrogenase A regulates tumor-macrophage symbiosis to promote glioblastoma progression. Nat Commun. (2024) 15:1987. doi: 10.1038/s41467-024-46193-z 38443336 PMC10914854

[20] Miller TE El Farran CA Couturier CP Chen Z D’Antonio JP Verga J . Programs, origins and immunomodulatory functions of myeloid cells in glioma. Nature. (2025) 640:1072–82. doi: 10.1038/s41586-025-08633-8 40011771 PMC12018266

[21] Abdelfattah N Kumar P Wang C Leu JS Flynn WF Gao R . Single-cell analysis of human glioma and immune cells identifies S100A4 as an immunotherapy target. Nat Commun. (2022) 13:767. doi: 10.1038/s41467-022-28372-y 35140215 PMC8828877

[22] DeCordova S Shastri A Tsolaki AG Yasmin H Klein L Singh SK . Molecular heterogeneity and immunosuppressive microenvironment in glioblastoma. Front Immunol. (2020) 11:1402. doi: 10.3389/fimmu.2020.01402 32765498 PMC7379131

[23] Mohan AA Tomaszewski WH Haskell-Mendoza AP Hotchkiss KM Singh K Reedy JL . Targeting immunometabolism in glioblastoma. Front Oncol. (2021) 11:696402. doi: 10.3389/fonc.2021.696402 34222022 PMC8242259

[24] Antonios JP Soto H Everson RG Moughon D Orpilla JR Shin NP . Immunosuppressive tumor-infiltrating myeloid cells mediate adaptive immune resistance via a PD-1/PD-L1 mechanism in glioblastoma. Neuro Oncol. (2017) 19:796–807. doi: 10.1093/neuonc/now287 28115578 PMC5464463

[25] Yang R Sun L Li CF Wang YH Yao J Li H . Galectin-9 interacts with PD-1 and TIM-3 to regulate T cell death and is a target for cancer immunotherapy. Nat Commun. (2021) 12:832. doi: 10.1038/s41467-021-21099-2 33547304 PMC7864927

[26] Lee C Yu D Kim HS Kim KS Chang CY Yoon HJ . Galectin-9 mediates the functions of microglia in the hypoxic brain tumor microenvironment. Cancer Res. (2024) 84:3788–802. doi: 10.1158/0008-5472.CAN-23-3878 39207402

[27] Xu W Dong J Zheng Y Zhou J Yuan Y Ta HM . Immune checkpoint protein VISTA regulates antitumor immunity by controlling myeloid cell–mediated inflammation and immunosuppression. Cancer Immunol Res. (2019) 7:1497–510. doi: 10.1158/2326-6066.CIR-18-0489 31340983 PMC6726548

[28] Qian J Luo F Yang J Liu J Liu R Wang L . TLR2 promotes glioma immune evasion by downregulating MHC class II molecules in microglia. Cancer Immunol Res. (2018) 6:1220–33. doi: 10.1158/2326-6066.CIR-18-0020 30131377

[29] von Roemeling CA Wang Y Qie Y Yuan H Zhao H Liu X . Therapeutic modulation of phagocytosis in glioblastoma can activate both innate and adaptive antitumour immunity. Nat Commun. (2020) 11:1508. doi: 10.1038/s41467-020-15129-8 32198351 PMC7083893

[30] Wang G Zhong K Tang X Tong A Zhou L . Tumor-associated microglia and macrophages in glioblastoma: from basic insights to therapeutic opportunities. Front Immunol. (2022) 13:964898. doi: 10.3389/fimmu.2022.964898 35967394 PMC9363573

[31] Zarodniuk M Steele A Lu X Li J Datta M . CNS tumor stroma transcriptomics identify perivascular fibroblasts as predictors of immunotherapy resistance in glioblastoma patients. NPJ Genom Med. (2023) 8:35. doi: 10.1038/s41525-023-00381-w 37884531 PMC10603041

[32] Watson SS Zomer A Fournier N Lourenco J Quadroni M Chryplewicz A . Fibrotic response to anti-CSF-1R therapy potentiates glioblastoma recurrence. Cancer Cell. (2024) 42:1507–1527.e11. doi: 10.1016/j.ccell.2024.08.012 39255775

[33] Koyama S Akbay EA Li YY Herter-Sprie GS Buczkowski KA Richards WG . Adaptive resistance to therapeutic PD-1 blockade is associated with upregulation of alternative immune checkpoints. Nat Commun. (2016) 7:10501. doi: 10.1038/ncomms10501 26883990 PMC4757784

[34] Wang M Jia J Cui Y Peng Y Jiang Y . CD73-positive extracellular vesicles promote glioblastoma immunosuppression by inhibiting T-cell clonal expansion. Cell Death Dis. (2021) 12:11. doi: 10.1038/s41419-021-04359-3 34753903 PMC8578373

[35] Chen Z Soni N Pinero G Giotti B Eddins DJ Lindblad KE . Monocyte depletion enhances neutrophil influx and proneural to mesenchymal transition in glioblastoma. Nat Commun. (2023) 14:1839. doi: 10.1038/s41467-023-37361-8 37012245 PMC10070461

[36] Schonfeld E Choi J Tran A Kim LH Lim M . The landscape of immune checkpoint inhibitor clinical trials in glioblastoma: a systematic review. Neuro Oncol Adv. (2024) 6:vdae174. doi: 10.1093/noajnl/vdae174 39534539 PMC11555435

[37] Zeng J See AP Phallen J Jackson CM Belcaid Z Ruzevick J . Anti-PD-1 blockade and stereotactic radiation produce long-term survival in mice with intracranial gliomas. Int J Radiat Oncol Biol Phys. (2013) 86:343–9. doi: 10.1016/j.ijrobp.2012.12.025 23462419 PMC3963403

[38] Kim JE Patel MA Mangraviti A Kim ES Theodros D Velarde E . Combination therapy with anti-PD-1, anti-TIM-3, and focal radiation results in regression of murine gliomas. Clin Cancer Res. (2017) 23:124–36. doi: 10.1158/1078-0432.CCR-15-1535 27358487 PMC5735836

[39] Sahebjam S Raval RR Forsyth PA Enderling H Tran ND Arrington JA . Phase 1 trial of hypofractionated stereotactic re-irradiation in combination with nivolumab, ipilimumab, and bevacizumab for recurrent high-grade gliomas. Neuro Oncol Adv. (2025) 7:vdaf033. doi: 10.1093/noajnl/vdaf033 40134851 PMC11934552

[40] Mathios D Kim JE Mangraviti A Phallen J Park CK Jackson CM . Anti-PD-1 antitumor immunity is enhanced by local and abrogated by systemic chemotherapy in GBM. Sci Transl Med. (2016) 8:370ra180. doi: 10.1126/scitranslmed.aag2942 28003545 PMC5724383

[41] Omuro A Reardon DA Sampson JH Baehring J Sahebjam S Cloughesy TF . Nivolumab plus radiotherapy with or without temozolomide in newly diagnosed glioblastoma: results from exploratory phase I cohorts of CheckMate 143. Neuro-Oncol Adv. (2022) 4:vdac025. doi: 10.1093/noajnl/vdac025 35402913 PMC8989388

[42] Xu W Dong J Zheng Y Zhou J Yuan Y Ta HM . Immune-checkpoint protein VISTA regulates antitumor immunity by controlling myeloid cell-mediated inflammation and immunosuppression. Cancer Immunol Res. (2019) 7:1497–510. doi: 10.1158/2326-6066.CIR-18-0489 31340983 PMC6726548

[43] Ye Z Ai X Yang K Yang Z Fei F Liao X . Targeting microglial metabolic rewiring synergizes with immune-checkpoint blockade therapy for glioblastoma. Cancer Discov. (2023) 13:974–1001. doi: 10.1158/2159-8290.CD-22-0455 36649564 PMC10073346

[44] Gabrusiewicz K Rodriguez B Wei J Hashimoto Y Healy LM Maiti SN . Glioblastoma-infiltrated innate immune cells resemble M0 macrophage phenotype. JCI Insight. (2016) 1. doi: 10.1172/jci.insight.85841 26973881 PMC4784261

[45] Ravi VM Neidert N Will P Joseph K Maier JP Kückelhaus J . T-cell dysfunction in the glioblastoma microenvironment is mediated by myeloid cells releasing interleukin-10. Nat Commun. (2022) 13:925. doi: 10.1038/s41467-022-28523-1 35177622 PMC8854421

[46] Lamano JB Lamano JB Li YD DiDomenico JD Choy W Veliceasa D . Glioblastoma-derived IL-6 induces immunosuppressive peripheral myeloid cell PD-L1 and promotes tumor growth. Clin Cancer Res. (2019) 25:3643–57. doi: 10.1158/1078-0432.CCR-18-2402 30824583 PMC6571046

[47] Weber R Riester Z Hüser L Sticht C Siebenmorgen A Groth C . IL-6 regulates CCR5 expression and immunosuppressive capacity of MDSC in murine melanoma. J Immunother Cancer. (2020) 8. doi: 10.1136/jitc-2020-000949 32788238 PMC7422659

[48] Weber R Groth C Lasser S Arkhypov I Petrova V Altevogt P . IL-6 as a major regulator of MDSC activity and possible target for cancer immunotherapy. Cell Immunol. (2021) 359:104254. doi: 10.1016/j.cellimm.2020.104254 33296753

[49] Tobin RP Jordan KR Kapoor P Spongberg E Davis D Vorwald VM . IL-6 and IL-8 are linked with myeloid-derived suppressor cell accumulation and correlate with poor clinical outcomes in melanoma patients. Front Oncol. (2019) 9:1223. doi: 10.3389/fonc.2019.01223 31781510 PMC6857649

[50] Yang F He Z Duan H Zhang D Li J Yang H . Synergistic immunotherapy of glioblastoma by dual targeting of IL-6 and CD40. Nat Commun. (2021) 12:3424. doi: 10.1038/s41467-021-23832-3 34103524 PMC8187342

[51] Parker S McDowall C Sanchez-Perez L Osorio C Duncker PC Briley A . Immunotoxin-αCD40 therapy activates innate and adaptive immunity and generates a durable antitumor response in glioblastoma models. Sci Transl Med. (2023) 15:eabn5649. doi: 10.1126/scitranslmed.abn5649 36753564 PMC10440725

[52] Akkari L Bowman RL Tessier J Klemm F Handgraaf SM de Groot M . Dynamic changes in glioma macrophage populations after radiotherapy reveal CSF-1R inhibition as a strategy to overcome resistance. Sci Transl Med. (2020) 12:eaaw7843. doi: 10.1126/scitranslmed.aaw7843 32669424

[53] Desgraves JF Mendez Valdez MJ Chandar J Gurses ME Henderson L Castro JR . Antisense oligonucleotides for rapid translation of gene therapy in glioblastoma. Cancers (Basel). (2024) 16:1944. doi: 10.3390/cancers16101944 38792022 PMC11119631

[54] Long GV Shklovskaya E Satgunaseelan L Mao Y da Silva IP Perry KA . Neoadjuvant triplet immune checkpoint blockade in newly diagnosed glioblastoma. Nat Med. (2025) 31:1–10. doi: 10.1038/s41591-025-03512-1 40016450 PMC12092302

[55] Venkatesh HS Morishita W Geraghty AC Silverbush D Gillespie SM Arzt M . Electrical and synaptic integration of glioma into neural circuits. Nature. (2019) 573:539–45. doi: 10.1038/s41586-019-1563-y 31534222 PMC7038898

[56] Venkataramani V Tanev DI Strahle C Studier-Fischer A Fankhauser L Kessler T . Glutamatergic synaptic input to glioma cells drives brain tumour progression. Nature. (2019) 573:532–8. doi: 10.1038/s41586-019-1564-x 31534219

[57] Krishna S Choudhury A Keough MB Seo K Ni L Kakaizada S . Glioblastoma remodelling of human neural circuits decreases survival. Nature. (2023) 617:599–607. doi: 10.1038/s41586-023-06036-1 37138086 PMC10191851

[58] Taylor KR Barron T Hui A Spitzer A Yalçin B Ivec AE . Glioma synapses recruit mechanisms of adaptive plasticity. Nature. (2023) 623:366–74. doi: 10.1038/s41586-023-06678-1 37914930 PMC10632140

[59] Venkatesh HS Tam LT Woo PJ Lennon J Nagaraja S Gillespie SM . Targeting neuronal activity-regulated neuroligin-3 dependency in high-grade glioma. Nature. (2017) 549:533–7. doi: 10.1038/nature24014 28959975 PMC5891832

[60] Lertsumitkul L Iliopoulos M Wang SS McArthur SJ Ebert LM Davenport AJ . EphA3-targeted chimeric antigen receptor T cells are effective in glioma and generate curative memory T cell responses. J Immunother Cancer. (2024) 12. doi: 10.1136/jitc-2024-009486 39111833 PMC11308882

[61] Kerhervé M Rosińska S Trillet K Zeinaty A Feyeux M Nedellec S . Neuropilin-1 modulates the 3D invasive properties of glioblastoma stem-like cells. Front Cell Dev Biol. (2022) 10:981583. doi: 10.3389/fcell.2022.981583 36204684 PMC9530787

[62] Sooreshjani M Tripathi S Dussold C Najem H De Groot J Lukas RV . The use of targeted cytokines as cancer therapeutics in glioblastoma. Cancers. (2023) 15:3739. doi: 10.3390/cancers15143739 37509400 PMC10378451

[63] Campian JL Ghosh S Kapoor V Yan R Thotala S Jash A . Long-acting recombinant human interleukin-7, NT-I7, increases cytotoxic CD8 T cells and enhances survival in mouse glioma models. Clin Cancer Res. (2022) 28:1229–39. doi: 10.1158/1078-0432.CCR-21-0947 35031547 PMC13020687

[64] Puri R Leland P Obiri N Husain S Kreitman R Haas G . Targeting of interleukin-13 receptor on human renal cell carcinoma cells by a recombinant chimeric protein composed of interleukin-13 and a truncated form of Pseudomonas exotoxin A (PE38QQR). Blood. (1996) 87:4333–9. doi: 10.1182/blood.V87.10.4333.bloodjournal87104333 8639793

[65] Rossmeisl JH Herpai D Quigley M Cecere TE Robertson JL D’Agostino RB . Phase I trial of convection-enhanced delivery of IL13RA2 and EPHA2 receptor targeted cytotoxins in dogs with spontaneous intracranial gliomas. Neuro-Oncology. (2021) 23:422–34. doi: 10.1093/neuonc/noaa196 32812637 PMC7992889

[66] Weiss T Puca E Silginer M Hemmerle T Pazahr S Bink A . Immunocytokines are a promising immunotherapeutic approach against glioblastoma. Sci Transl Med. (2020) 12:eabb2311. doi: 10.1126/scitranslmed.abb2311 33028706

[67] Look T Puca E Bühler M Kirschenbaum D De Luca R Stucchi R . Targeted delivery of tumor necrosis factor in combination with CCNU induces a T cell–dependent regression of glioblastoma. Sci Transl Med. (2023) 15:eadf2281. doi: 10.1126/scitranslmed.adf2281 37224228

[68] Mohme M Schliffke S Maire CL Rünger A Glau L Mende KC . Immunophenotyping of newly diagnosed and recurrent glioblastoma defines distinct immune exhaustion profiles in peripheral and tumor-infiltrating lymphocytes. Clin Cancer Res. (2018) 24:4187–200. doi: 10.1158/1078-0432.CCR-17-2617 29444930

[69] Chiu DKC Zhang X Cheng BYL Liu Q Hayashi K Yu B . Tumor-derived erythropoietin acts as an immunosuppressive switch in cancer immunity. Science. (2025) 388:eadr3026. doi: 10.1126/science.adr3026 40273234 PMC12110762

[70] Chen KS Reinshagen C Van Schaik TA Rossignoli F Borges P Mendonca NC . Bifunctional cancer cell–based vaccine concomitantly drives direct tumor killing and antitumor immunity. Sci Transl Med. (2023) 15:eabo4778. doi: 10.1126/scitranslmed.abo4778 36599004 PMC10068810

[71] Meister H Look T Roth P Pascolo S Sahin U Lee S . Multifunctional mRNA-based CAR T cells display promising antitumor activity against glioblastoma. Clin Cancer Res. (2022) 28:4747–56. doi: 10.1158/1078-0432.CCR-21-4384 36037304

[72] Birocchi F Cusimano M Rossari F Beretta S Rancoita PMV Ranghetti A . Targeted inducible delivery of immunoactivating cytokines reprograms glioblastoma microenvironment and inhibits growth in mouse models. Sci Transl Med. (2022) 14:eabl4106. doi: 10.1126/scitranslmed.abl4106 35857642

[73] Van Schaik TA Chen KS Kanaya N Moreno-Lama L Freeman NW Wang M . Antitumor immunity mediated by engineered stem cells exploiting TRAIL-induced cell death and FLT3L immunomodulation. Clin Cancer Res. (2025) 31:2793–813. doi: 10.1158/1078-0432.CCR-24-3835 40238542

[74] Montoya M Collins SA Chuntova P Patel TS Nejo T Yamamichi A . Interferon regulatory factor 8-driven reprogramming of the immune microenvironment enhances antitumor adaptive immunity and reduces immunosuppression in murine glioblastoma. Neuro-Oncology. (2024) 26:2272–87. doi: 10.1093/neuonc/noae149 39115195 PMC11630541

[75] Tian L Xu B Chen Y Li Z Wang J Zhang J . Specific targeting of glioblastoma with an oncolytic virus expressing a cetuximab-CCL5 fusion protein via innate and adaptive immunity. Nat Cancer. (2022) 3:1318–35. doi: 10.1038/s43018-022-00448-0 36357700 PMC10150871

[76] Ahluwalia MS Peereboom DM Ciolfi M Schilero C Hobbs B Ciesielski MJ . Phase II study of pembrolizumab plus SurVaxM for glioblastoma at first recurrence. JCO. (2020) 38:TPS2581–TPS2581. doi: 10.1200/JCO.2020.38.15_suppl.TPS2581 37530309

[77] Peereboom D . Study of Pembrolizumab Plus SurVaxM for Glioblastoma at First Recurrence (2024). Available online at: https://clinicaltrials.gov/study/NCT04013672?tab=table (Accessed February 23, 2026).

[78] Lazarus H Ragsdale C Gale R Lyman G . Sargramostim (rhu GM-CSF) as cancer therapy (systematic review) and an immunomodulator. A drug before its time? Front Immunol. (2021) 12:706186. doi: 10.3389/fimmu.2021.706186 34484202 PMC8416151

[79] Khasraw M . Immunotherapy Targeted Against Cytomegalovirus in Patients With Newly-Diagnosed WHO Grade IV Unmethylated Glioma (I-ATTAC) (2024). Available online at: https://clinicaltrials.gov/study/NCT03927222 (Accessed February 23, 2026).

[80] Safety and Efficacy Study of SL-701, a Glioma-Associated Antigen Vaccine To Treat Recurrent Glioblastoma Multiforme (2025). Available online at: https://clinicaltrials.gov/study/NCT02078648?cond=%22Glioma%22&intr=%22Cathartics%22&viewType=Table&rank=6 (Accessed February 23, 2026).

[81] Salazar A Levy H Ondra S Kende M Scherokman B Brown D . Long-term treatment of Malignant gliomas with intramuscularly administered polyinosinic-polycytidylic acid stabilized with polylysine and carboxymethylcellulose: An open pilot study. Neurosurgery. (1996) 38:1096–104. doi: 10.1227/00006123-199606000-00006 8727138

[82] National Cancer Institute (NCI) . CAR T Cell Receptor Immunotherapy Targeting EGFRvIII for Patients With Malignant Gliomas Expressing EGFRvIII (2019). Available online at: https://clinicaltrials.gov/study/NCT01454596?tab=results (Accessed February 23, 2026).

[83] Amaria R Reuben A Cooper Z Wargo J . Update on use of aldesleukin for treatment of high-risk metastatic melanoma. ITT. (2015) 4:79. doi: 10.2147/ITT.S61590 27471714 PMC4918260

[84] Goff SL Morgan RA Yang JC Sherry RM Robbins PF Restifo NP . Pilot trial of adoptive transfer of chimeric antigen receptor–transduced T cells targeting EGFRvIII in patients with glioblastoma. J Immunother. (2019) 42:126–35. doi: 10.1097/CJI.0000000000000260 30882547 PMC6691897

[85] Sampson JH Singh Achrol A Aghi MK Bankiewicz K Bexon M Brem S . Targeting the IL4 receptor with MDNA55 in patients with recurrent glioblastoma: Results of a phase IIb trial. Neuro-Oncology. (2023) 25:1085–97. doi: 10.1093/neuonc/noac285 36640127 PMC10237418

[86] Medicenna Therapeutics, Inc . Convection-Enhanced Delivery (CED) of MDNA55 in Adults With Recurrent or Progressive Glioblastoma (2022). Available online at: https://clinicaltrials.gov/study/NCT02858895?tab=results (Accessed February 23, 2026).

[87] Faust Akl C Andersen BM Li Z Giovannoni F Diebold M Sanmarco LM . Glioblastoma-instructed astrocytes suppress tumour-specific T cell immunity. Nature. (2025) 643:219–29. doi: 10.1038/s41586-025-08997-x 40399681 PMC12765214

[88] Ozaki K Leonard W . Cytokine and cytokine receptor pleiotropy and redundancy. J Biol Chem. (2002) 277:29355–8. doi: 10.1074/jbc.R200003200 12072446

[89] Neri D Sondel P . Immunocytokines for cancer treatment: past, present and future. Curr Opin Immunol. (2016) 40:96–102. doi: 10.1016/j.coi.2016.03.006 27060634 PMC5215124

[90] Yuan F Wang Y Yuan L Tang T Ye L Li Y . EGFRvIII-positive glioblastoma contributes to immune escape and Malignant progression via the c-Fos-MDK-LRP1 axis. Cell Death Dis. (2025) 16:453. doi: 10.1038/s41419-025-07771-1 40527884 PMC12174314

[91] Goff SL Morgan RA Yang JC Sherry RM Robbins PF Restifo NP . Pilot trial of adoptive transfer of chimeric antigen receptor-transduced T cells targeting EGFRvIII in patients with glioblastoma. J Immunother. (2019) 42:126–35. doi: 10.1097/CJI.0000000000000260 30882547 PMC6691897

[92] Bagley SJ Binder ZA Lamrani L Marinari E Desai AS Nasrallah MP . Repeated peripheral infusions of anti-EGFRvIII CAR T cells in combination with pembrolizumab show no efficacy in glioblastoma: a phase 1 trial. Nat Cancer. (2024) 5:517–31. doi: 10.1038/s43018-023-00709-6 38216766

[93] Mineo JF Bordron A Baroncini M Maurage CA Ramirez C Siminski RM . Low HER2-expressing glioblastomas are more often secondary to anaplastic transformation of low-grade glioma. J Neuro-Oncol. (2007) 85:281–7. doi: 10.1007/s11060-007-9424-1 17571214

[94] Zeng J Zhang J Yang YZ Wang F Jiang H Chen HD . IL13RA2 is overexpressed in Malignant gliomas and related to clinical outcome of patients. Am J Transl Res. (2020) 12:4702–14. PMC7476143 32913543

[95] Brown CE Badie B Barish ME Weng L Ostberg JR Chang WC . Bioactivity and safety of IL13Rα2-redirected chimeric antigen receptor CD8+ T cells in patients with recurrent glioblastoma. Clin Cancer Res. (2015) 21:4062–72. doi: 10.1158/1078-0432.CCR-15-0428 26059190 PMC4632968

[96] Brown CE Hibbard JC Alizadeh D Blanchard MS Natri HM Wang D . Locoregional delivery of IL-13Rα2-targeting CAR-T cells in recurrent high-grade glioma: a phase 1 trial. Nat Med. (2024) 30:1001–12. doi: 10.1038/s41591-024-02875-1 38454126 PMC11031404

[97] O’Rourke DM Nasrallah MP Desai A Melenhorst JJ Mansfield K Morrissette JJD . A single dose of peripherally infused EGFRvIII-directed CAR T cells mediates antigen loss and induces adaptive resistance in patients with recurrent glioblastoma. Sci Transl Med. (2017) 9. doi: 10.1126/scitranslmed.aaa0984 28724573 PMC5762203

[98] Choe JH Watchmaker PB Simic MS Gilbert RD Li AW Krasnow NA . SynNotch-CAR T cells overcome challenges of specificity, heterogeneity, and persistence in treating glioblastoma. Sci Transl Med. (2021) 13. doi: 10.1126/scitranslmed.abe7378 33910979 PMC8362330

[99] Choi BD Gerstner ER Frigault MJ Leick MB Mount CW Balaj L . Intraventricular CARv3-TEAM-E T cells in recurrent glioblastoma. N Engl J Med. (2024) 390:1290–8. doi: 10.1056/NEJMoa2314390 38477966 PMC11162836

[100] Schmidts A Srivastava AA Ramapriyan R Bailey SR Bouffard AA Cahill DP . Tandem chimeric antigen receptor (CAR) T cells targeting EGFRvIII and IL-13Rα2 are effective against heterogeneous glioblastoma. Neuro-Oncol Adv. (2023) 5:vdac185. doi: 10.1093/noajnl/vdac185 36751672 PMC9896600

[101] Bagley SJ Logun M Fraietta JA Wang X Desai AS Bagley LJ . Intrathecal bivalent CAR T cells targeting EGFR and IL13Rα2 in recurrent glioblastoma: phase 1 trial interim results. Nat Med. (2024) 30:1320–9. doi: 10.1038/s41591-024-02893-z 38480922 PMC13123313

[102] Ronsley R Choe M Seidel K Narayanaswamy P Pattabhi S Wendler J . IMMU-01. Pioneering quad-targeting CAR T cell therapy in pediatric CNS tumors – analysis from the initial patients treated on the first-in-human phase 1 trial BRAINCHILD-04. Neuro-Oncology. (2024) 26. doi: 10.1093/neuonc/noae064.372 36757281

[103] De Sostoa J Marinari E Pedard M Widmer V Davanture S Schaller K . Targeting the extracellular matrix with Tenascin-C-specific CAR T cells extends survival in preclinical models of glioblastoma. J Immunother Cancer. (2025) 13:e011382. doi: 10.1136/jitc-2024-011382 41188009 PMC12593508

[104] Shanley M Daher M Dou J Li S Basar R Rafei H . Interleukin-21 engineering enhances NK cell activity against glioblastoma via CEBPD. Cancer Cell. (2024) 42:1450–1466.e11. doi: 10.1016/j.ccell.2024.07.007 39137729 PMC11370652

[105] Burger MC Forster MT Romanski A Straßheimer F Macas J Zeiner PS . Intracranial injection of natural killer cells engineered with a HER2-targeted chimeric antigen receptor in patients with recurrent glioblastoma. Neuro Oncol. (2023) 25:2058–71. doi: 10.1093/neuonc/noad087 37148198 PMC10628939

[106] Khagi S Carrillo J Park D Drusbosky L Bhar P Zhang H . CTIM-23. First-in-human immunotherapy targeting lymphopenia in recurrent glioblastoma multiform (GBM): NAI and PD-L1 T-HANK plus bevacizumab with complete response. Neuro-Oncology. (2025) 27:v119–9. doi: 10.1093/neuonc/noaf201.0480 36757281

[107] Lupo KB Yao X Borde S Wang J Torregrosa-Allen S Elzey BD . synNotch-programmed iPSC-derived NK cells usurp TIGIT and CD73 activities for glioblastoma therapy. Nat Commun. (2024) 15:1. doi: 10.1038/s41467-024-46343-3 38429294 PMC10907695

[108] Look T Sankowski R Bouzereau M Fazio S Sun M Buck A . CAR T cells, CAR NK cells, and CAR macrophages exhibit distinct traits in glioma models but are similarly enhanced when combined with cytokines. CR Med. (2025) 6. doi: 10.1016/j.xcrm.2025.101931 39889712 PMC11866521

[109] Li YR Zhu Y Li Z Shen X Halladay T Tse C . Allogeneic stem cell-engineered EGFRvIII-specific CAR-NKT cells for treating glioblastoma with enhanced efficacy and safety. Mol Ther. (2025) 33:6041–62. doi: 10.1016/j.ymthe.2025.09.026 40946163 PMC12703166

[110] Kang I Kim Y Lee HK . γδ T cells as a potential therapeutic agent for glioblastoma. Front Immunol. (2023) 14:1273986. doi: 10.3389/fimmu.2023.1273986 37928546 PMC10623054

[111] Pant A Lim M . CAR-T therapy in GBM: Current challenges and avenues for improvement. Cancers (Basel). (2023) 15. doi: 10.3390/cancers15041249 36831591 PMC9954019

[112] Strassheimer F Elleringmann P Ludmirski G Roller B Macas J Alekseeva T . CAR-NK cell therapy combined with checkpoint inhibition induces an NKT cell response in glioblastoma. Br J Cancer. (2025) 132:849–60. doi: 10.1038/s41416-025-02977-8 40102596 PMC12041480

[113] Martins TA Kaymak D Tatari N Gerster F Hogan S Ritz MF . Enhancing anti-EGFRvIII CAR T cell therapy against glioblastoma with a paracrine SIRPγ-derived CD47 blocker. Nat Commun. (2024) 15:9718. doi: 10.1038/s41467-024-54129-w 39521782 PMC11550474

[114] Wang J Toregrosa-Allen S Elzey BD Utturkar S Lanman NA Bernal-Crespo V . Multispecific targeting of glioblastoma with tumor microenvironment-responsive multifunctional engineered NK cells. Proc Natl Acad Sci. (2021) 118:e2107507118. doi: 10.1073/pnas.2107507118 34740973 PMC8609337

[115] Adachi K Kano Y Nagai T Okuyama N Sakoda Y Tamada K . IL-7 and CCL19 expression in CAR-T cells improves immune cell infiltration and CAR-T cell survival in the tumor. Nat Biotechnol. (2018) 36:346–51. doi: 10.1038/nbt.4086 29505028

[116] Luo H Su J Sun R Sun Y Wang Y Dong Y . Coexpression of IL7 and CCL21 increases efficacy of CAR-T cells in solid tumors without requiring preconditioned lymphodepletion. Clin Cancer Res. (2020) 26:5494–505. doi: 10.1158/1078-0432.CCR-20-0777 32816947

[117] Hou AJ Chang ZL Lorenzini MH Zah E Chen YY . TGF-β-responsive CAR-T cells promote anti-tumor immune function. Bioeng Transl Med. (2018) 3:75–86. doi: 10.1002/btm2.10097 30065964 PMC6063867

[118] Neftel C Laffy J Filbin MG Hara T Shore ME Rahme GJ . An integrative model of cellular states, plasticity, and genetics for glioblastoma. Cell. (2019) 178:835–849.e21. doi: 10.1016/j.cell.2019.06.024 31327527 PMC6703186

[119] Becker AP Sells BE Haque SJ Chakravarti A . Tumor heterogeneity in glioblastomas: From light microscopy to molecular pathology. Cancers (Basel). (2021) 13:761. doi: 10.3390/cancers13040761 33673104 PMC7918815

[120] XieZ JanczykPŁ CornelisonR LynchS Glowczyk-GlucM LeiferB . A first-in-class small-molecule inhibitor targeting AVIL exhibits safety and antitumor efficacy in preclinical models of glioblastoma. Sci Transl Med. (2026) 18:eadt1211. doi: 10.1126/scitranslmed.adt1211 41604465

[121] Logun M Wang X Sun Y Bagley SJ Li N Desai A . Patient-derived glioblastoma organoids as real-time avatars for assessing responses to clinical CAR-T cell therapy. Cell Stem Cell. (2025) 32:181–190.e4. doi: 10.1016/j.stem.2024.11.010 39657679 PMC11808387

[122] Schuster J Lai RK Recht LD Reardon DA Paleologos NA Groves MD . A phase II, multicenter trial of rindopepimut (CDX-110) in newly diagnosed glioblastoma: The ACT III study. Neuro Oncol. (2015) 17:854–61. doi: 10.1093/neuonc/nou348 25586468 PMC4483122

[123] Weller M Butowski N Tran DD Recht LD Lim M Hirte H . Rindopepimut with temozolomide for patients with newly diagnosed, EGFRvIII-expressing glioblastoma (ACT IV): A randomised, double-blind, international phase 3 trial. Lancet Oncol. (2017) 18:1373–85. doi: 10.1016/S1470-2045(17)30517-X 28844499

[124] Reardon DA Desjardins A Vredenburgh JJ O’Rourke DM Tran DD Fink KL . Rindopepimut with bevacizumab for patients with relapsed EGFRvIII-expressing glioblastoma (ReACT): Results of a double-blind randomized phase II trial. Clin Cancer Res. (2020) 26:1586–94. doi: 10.1158/1078-0432.CCR-18-1140 32034072

[125] Ahluwalia MS Reardon DA Abad AP Curry WT Wong ET Figel SA . Phase IIa study of SurVaxM plus adjuvant temozolomide for newly diagnosed glioblastoma. J Clin Oncol. (2023) 41:1453–65. doi: 10.1200/JCO.22.00996 36521103 PMC9995096

[126] Liau LM Ashkan K Brem S Campian JL Trusheim JE Iwamoto FM . Association of autologous tumor lysate-loaded dendritic cell vaccination with extension of survival among patients with newly diagnosed and recurrent glioblastoma: A phase 3 prospective externally controlled cohort trial. JAMA Oncol. (2023) 9:112–21. doi: 10.1001/jamaoncol.2022.5370 36394838 PMC9673026

[127] Hilf N Kuttruff-Coqui S Frenzel K Bukur V Stevanović S Gouttefangeas C . Actively personalized vaccination trial for newly diagnosed glioblastoma. Nature. (2019) 565:240–5. doi: 10.1038/s41586-018-0810-y 30568303

[128] Mendez-Gomez HR DeVries A Castillo P von Roemeling C Qdaisat S Stover BD . RNA aggregates harness the danger response for potent cancer immunotherapy. Cell. (2024) 187:10. doi: 10.1016/j.cell.2024.04.003 38697107 PMC11767857

[129] Piranlioglu R Chiocca EA . Oncolytic virus-mediated immunomodulation in glioblastoma: Insights from clinical trials and challenges. Semin Immunol. (2025) 79:101975. doi: 10.1016/j.smim.2025.101975 40555086 PMC12878671

[130] Cheema TA Wakimoto H Fecci PE Ning J Kuroda T Jeyaretna DS . Multifaceted oncolytic virus therapy for glioblastoma in an immunocompetent cancer stem cell model. Proc Natl Acad Sci. (2013) 110:12006–11. doi: 10.1073/pnas.1307935110 23754388 PMC3718117

[131] Saha D Martuza RL Rabkin SD . Macrophage polarization contributes to glioblastoma eradication by combination immunovirotherapy and immune checkpoint blockade. Cancer Cell. (2017) 32:253–267.e5. doi: 10.1016/j.ccell.2017.07.006 28810147 PMC5568814

[132] Todo T Ito H Ino Y Ohtsu H Ota Y Shibahara J . Intratumoral oncolytic herpes virus G47Δ for residual or recurrent glioblastoma: A phase 2 trial. Nat Med. (2022) 28:1630–9. doi: 10.1038/s41591-022-01897-x 35864254 PMC9388376

[133] Lang FF Conrad C Gomez-Manzano C Yung WKA Sawaya R Weinberg JS . Phase I study of DNX-2401 (Delta-24-RGD) oncolytic adenovirus: Replication and immunotherapeutic effects in recurrent Malignant glioma. J Clin Oncol. (2018) 36:1419–27. doi: 10.1200/JCO.2017.75.8219 29432077 PMC6075856

[134] Ribas A Dummer R Puzanov I VanderWalde A Andtbacka RHI Michielin O . Oncolytic virotherapy promotes intratumoral T cell infiltration and improves anti-PD-1 immunotherapy. Cell. (2017) 170:1109–1119.e10. doi: 10.1016/j.cell.2017.08.027 28886381 PMC8034392

[135] Nassiri F Patil V Yefet LS Singh O Liu J Dang RMA . Oncolytic DNX-2401 virotherapy plus pembrolizumab in recurrent glioblastoma: A phase 1/2 trial. Nat Med. (2023) 29:6. doi: 10.1038/s41591-023-02347-y 37188783 PMC10287560

[136] Ling AL Solomon IH Landivar AM Nakashima H Woods JK Santos A . Clinical trial links oncolytic immunoactivation to survival in glioblastoma. Nature. (2023) 623:157–66. doi: 10.1038/s41586-023-06623-2 37853118 PMC10620094

[137] Ling AL Gantchev J Prabhu MC Basu S Ahn R D’Souza A . Serial multiomics uncovers anti-glioblastoma responses not evident by routine clinical analyses. Sci Transl Med. (2025) 17:eadv2881. doi: 10.1126/scitranslmed.adv2881 41061048 PMC13574239

[138] Przystal JM Becker H Canjuga D Tsiami F Anderle N Keller AL . Targeting CSF1R alone or in combination with PD1 in experimental glioma. Cancers. (2021) 13:10. doi: 10.3390/cancers13102400 34063518 PMC8156558

[139] Butowski NA Colman H De Groot JF Omuro AMP Nayak L Cloughesy TF . A phase 2 study of orally administered PLX3397 in patients with recurrent glioblastoma. J Clin Oncol. (2014) 32:2023. doi: 10.1200/jco.2014.32.15_suppl.2023

[140] Nomura M Spitzer A Johnson KC Garofano L Nehar-belaid D Galili Darnell N . The multilayered transcriptional architecture of glioblastoma ecosystems. Nat Genet. (2025) 57:1155–67. doi: 10.1038/s41588-025-02167-5 40346361 PMC12081307

[141] Savage WM Yeary MD Tang AJ Sperring CP Argenziano MG Adapa AR . Biomarkers of immunotherapy in glioblastoma. Neuro-Oncol Pract. (2024) 11:383–94. doi: 10.1093/nop/npae028 39006524 PMC11241363

